# Multiscale modelling of drug transport and metabolism in liver spheroids

**DOI:** 10.1098/rsfs.2019.0041

**Published:** 2020-02-14

**Authors:** Joseph A. Leedale, Jonathan A. Kyffin, Amy L. Harding, Helen E. Colley, Craig Murdoch, Parveen Sharma, Dominic P. Williams, Steven D. Webb, Rachel N. Bearon

**Affiliations:** 1 EPSRC Liverpool Centre for Mathematics in Healthcare, Department of Mathematical Sciences, University of Liverpool, Liverpool L69 7ZL, UK; 2 Department of Applied Mathematics, Liverpool John Moores University, Liverpool L3 3AF, UK; 3 School of Clinical Dentistry, University of Sheffield, Claremont Crescent, Sheffield S10 2TA, UK; 4 MRC Centre for Drug Safety Science, Department of Molecular and Clinical Pharmacology, University of Liverpool, Liverpool L69 3GE, UK; 5 AstraZeneca, IMED Biotech Unit, Drug Safety and Metabolism, Cambridge Science Park, Cambridge CB4 0FZ, UK

**Keywords:** drug transport, spheroid, organoid, hepatocytes, systems pharmacology, mathematical modelling

## Abstract

In early preclinical drug development, potential candidates are tested in the laboratory using isolated cells. These *in vitro* experiments traditionally involve cells cultured in a two-dimensional monolayer environment. However, cells cultured in three-dimensional spheroid systems have been shown to more closely resemble the functionality and morphology of cells *in vivo*. While the increasing usage of hepatic spheroid cultures allows for more relevant experimentation in a more realistic biological environment, the underlying physical processes of drug transport, uptake and metabolism contributing to the spatial distribution of drugs in these spheroids remain poorly understood. The development of a multiscale mathematical modelling framework describing the spatio-temporal dynamics of drugs in multicellular environments enables mechanistic insight into the behaviour of these systems. Here, our analysis of cell membrane permeation and porosity throughout the spheroid reveals the impact of these properties on drug penetration, with maximal disparity between zonal metabolism rates occurring for drugs of intermediate lipophilicity. Our research shows how mathematical models can be used to simulate the activity and transport of drugs in hepatic spheroids and in principle any organoid, with the ultimate aim of better informing experimentalists on how to regulate dosing and culture conditions to more effectively optimize drug delivery.

## 1. Introduction

The discovery of potential toxicity *in vitro* remains an important process in providing preclinical safety assurances during drug development. However, conventional two-dimensional *in vitro* experiments, such as monolayer cell culture, tend to be poorly predictive of toxicity, and emerging three-dimensional systems are shown to be more physiologically relevant and predictive of the *in vivo* environment [1,2]. Accordingly, three-dimensional cell culture systems such as multicellular spheroids are increasingly being used in drug development and hepatic safety assessment [3,4]*.* Although three-dimensional spheroid systems offer improvements in terms of physiological relevance and *in vivo*-like functionality, the mechanistic interaction between these systems and drugs is not yet fully understood.

Multiscale *in silico* methods can improve the application of three-dimensional spheroid models to assess the hepatotoxicity of drug candidates [5,6]. Indeed, mechanistic mathematical modelling of drug metabolism and transport in three-dimensional microtissues is important for the pharmaceutical industry as it facilitates an improved platform for both preclinical drug development and *in vivo* extrapolation [7]. This utilization of mathematical models, devised to tackle pharmacological research challenges in a systems biology approach, has become known as part of the evolving field(s) of systems pharmacology and/or systems toxicology [8,9]. This approach is a multiscale, multidisciplinary field that employs holistic, integrative methods in order to enhance the understanding and prediction of emergent system properties. Moreover, this methodology is strictly quantitative requiring the integration of quantitative data and modelling to develop mechanistic knowledge of the system and reveal pharmacological and toxicological properties. Consequently, systems pharmacology models are becoming an increasingly important part of the toolkit to improve capabilities and drive innovation for *in vitro* safety assessment [10–12].

In this study, we have characterized the spatio-temporal dynamics of drugs in an *in vitro* hepatic spheroid system by simulating relevant physical processes *in silico*. A data-driven, multiscale, mathematical modelling framework combining mechanistic information relating to the diffusion, transport and metabolism of chemical species in a hepatocyte spheroid is presented. A microscale single-cell model is analysed to study different transport mechanisms by varying boundary conditions on the cell membrane. This model is then coupled to a multicellular model developed to evaluate the effects of cellular arrangement and density on the transport and penetration of drugs, simulating the *in vitro* microtissue environment. Such effects include a nonlinear relationship between drug lipophilicity and spheroid penetration, whereby drug delivery to the spheroid core is minimized for drugs of intermediate lipophilicity. The integration of experimental data allows for the development of realistic geometries and parametrization of the multiscale model for a range of drugs. Ultimately, by accurately simulating the processes of drug transport and metabolism we aim to enhance the understanding of underlying mechanisms and optimize the use of these systems *in vitro*.

## 2. Material and Methods

### 2.1. Microscale transport: crossing the cell membrane

**Figure 1.**
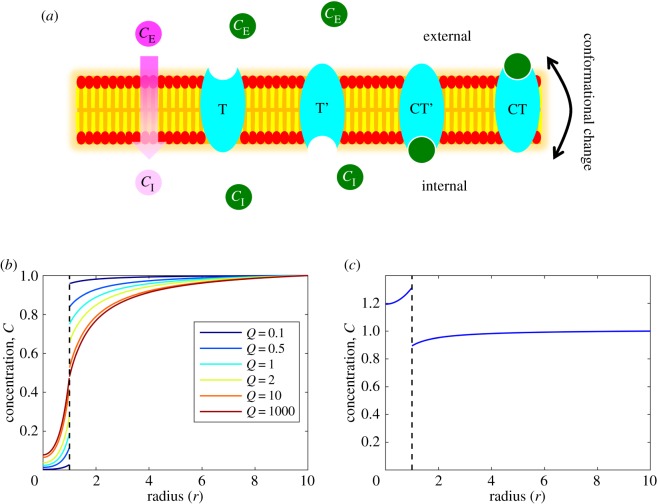
Modelling transmembrane transport in a single cell. (*a*) Drug transport schematics across the cell membrane. Two modes of transport are considered, passive diffusion (pink substrates/circles) and carrier-mediated transport (green substrates/circles). Drugs that permeate the cell via passive diffusion move down a concentration gradient directly through the membrane and are limited by their associated permeability coefficient. This coefficient is dependent on the physico-chemical properties of the drug, and drugs which cross the membrane via this mechanism are typically small and lipophilic. Other drugs may require the action of specific membrane-bound transporter proteins to enter the cell (carrier-mediated transport). In this study, it is assumed that this mechanism is dependent on carrier proteins/receptors (depicted in cyan) which can reversibly bind to the substrate and undergo conformational changes to transport the substrate across the membrane. Species within the figure are annotated with symbols related to mathematical models described in the main text and supplementary material. (*b*) Variation in the permeability coefficient determines the steady-state concentration profile of drug concentration in a single cell for the passive diffusion transport mechanism. Low permeability results in a discontinuity at the cell membrane $\left(D=2,V_{\max}=10,K_{m}=0.5,C_{r_{\max}}=1\right)$. (*c*) For specific parameter choices within the carrier-mediated transport model, a steady-state can be reached such that the drug is transported against its concentration gradient, implicitly simulating an active process $\left(D=2,V_{\max}=1,K_{m}=0.5,C_{r_{\max}}=1,T_{0}=1,\alpha_{1}=0.5,\alpha_{2}=1,\alpha_{3}=0,\alpha_{4}=0,\alpha_{5}=0\right)$. Full spatio-temporal dynamics can be found for (*b*) and (*c*) in supplementary animations.

To simulate the distribution of drugs throughout a three-dimensional tissue comprising multiple hepatocytes, it is necessary to determine how drugs penetrate and cross the cell membrane. This membrane comprises a phospholipid bilayer, providing a hydrophobic protective barrier for the cell. Consequently, this chemical barrier property is a key determinant in the effective permeability of any drug. Many factors affect drug permeability in tissues such as ionization, aqueous diffusion between lipid barriers and protein binding, but the partition into the membrane (determined by lipid solubility) is one of the most important [13]. Highly lipophilic substances can more readily penetrate the membrane via free diffusion, while relatively hydrophilic substances (highly soluble in polar solvents such as water or blood) cannot enter the cell easily and require specific transporters (figure 1*a*). The relative role of transporter proteins in intracellular drug transport is still debated and there remain different views as to whether passive diffusion or carrier-mediated transport is the major mechanism [14–18]. For the entirety of this study, we refer to the two main types of transport: passive diffusion—entering cells down a concentration gradient directly through the membrane (passive) and carrier-mediated transport—entering cells via specific transporter proteins embedded in the plasma membrane (passive or active).

The mathematical representation of microscale drug transport across a cell membrane can be studied with a simple model considering the processes governing drug concentration dynamics in two phases, inside and outside the cell, with a permeable barrier in between. Once inside the cell, the drug is removed via metabolism. We assume diffusion occurs at different rates inside (*D*_I_) and outside (*D*_E_) the cell, which we initially assume is spherical of radius *R*, but relax this assumption in §2.3. The drug concentration (*C*) dynamics inside the cell are given by the partial differential equation (PDE)

2.1 $$\frac{\partial C}{\partial t}=D_{I}\nabla^{2}C-\frac{V_{\max}C}{C+K_{m}},$$

where *V*_max_ is the maximum metabolic rate and *K*_m_ represents the drug concentration at which metabolism is half maximal. Since there is no flow within the *in vitro* system, and the dominant form of removal within the multiscale model is assumed to be due to intracellular metabolism, we assume that outside the cell drug transport is governed by diffusion processes only

2.2 $$\frac{\partial C}{\partial t}=D_{E}\nabla^{2}C.$$

For simplicity, we assume that the problem is radially symmetric and rescale the model with respect to cell radius and internal diffusion time (such that the cell boundary is now given by *r* = 1) to give

2.3 $$\frac{\partial C}{\partial t}=\frac{1}{r^{2}}\frac{\partial }{\partial r}\left(r^{2}\frac{\partial C}{\partial r}\right)-\frac{V_{\max}C}{C+K_{m}},\;r\leq 1\;$$

and

2.4 $$\frac{\partial C}{\partial t}=\frac{D}{r^{2}}\frac{\partial }{\partial r}\left(r^{2}\frac{\partial C}{\partial r}\right),\;r>1,$$

where *D* = *D*_E_/*D*_I_ due to rescaling (see supplementary material for details). We impose the following boundary conditions at the cell centre (*r* = 0), for radial symmetry, and a distance away from the cell (*r* = *r*_max_)

2.5 $$\frac{\partial C}{\partial r}=0,\;r=0\;$$

and

2.6 $$C=C_{r_{\max}},\;r=r_{\max},$$

where $C_{r_{\max}}$ is a constant supply term. Assume that the flux at the cell boundary is equal such that mass is conserved, i.e.

2.7 $$D_{I}\frac{\partial C_{I}}{\partial r}=D_{E}\frac{\partial C_{E}}{\partial r},\;r=1,$$

where *C*_I_ and *C*_E_ are used to distinguish between interior and exterior drug concentrations at the cell membrane boundary. A further boundary condition must be specified at the cell membrane boundary in order to solve the coupled PDE system and investigate the effects of different means of drug transport.

#### 2.1.1. Passive diffusion

The following boundary condition is imposed to describe the flux of drug into the cell due to passive diffusion:

2.8 $$D_{I}\frac{\partial C_{I}}{\partial r}=D_{E}\frac{\partial C_{E}}{\partial r}=Q(C_{E}-C_{I}),\;r=1,$$

where *Q* is the permeability coefficient. The mathematical model can be solved numerically in Matlab R2017b. For methodological details regarding derivations, numerical solutions and simulations of microscale transport, see the electronic supplementary material.

The impact of the permeability coefficient, *Q*, on the steady-state distribution of drug concentration can be seen in figure 1*b* (for temporal dynamics, see electronic supplementary animations). For low permeability coefficients (*Q* ≪ 1), there is less drug penetration per unit time and so there is a low steady-state value inside the cell and a large discontinuity at the cell membrane. As *Q* increases, relatively more drug enters the cell per unit time and in the limit, as *Q* → ∞, the steady-state solutions converge such that the drug concentration profile is continuous (*C*_E_ = *C*_I_) at the cell membrane boundary (which now provides no effective barrier or resistance) and the steady-state profile represents the balance of supply via diffusion and removal via intracellular metabolism.

#### 2.1.2. Carrier-mediated transport

For drugs whose physico-chemical properties prohibit direct permeation across the cell membrane, specific transporter proteins are required that can mediate the transfer process. The reliance on transporter (or carrier) proteins dictates that the flux is now saturable with an explicit dependence on the surface area concentration, binding affinities and activity of transporters in the cell membrane. In this scenario, the boundary condition representing membrane transport cannot be sufficiently represented by the passive diffusion condition in equation (2.8) and so we implement a simple carrier model as applied in other similar physiological membrane transport models, e.g. Keener & Sneyd [19] and Wood & Whitaker [20]. This carrier model can be applied to define the flux boundary condition for the carrier-mediated transport model scenario

2.9 $$D_{I}\frac{\partial C_{I}}{\partial r}=\frac{T_{0}(C_{E}\;-\alpha_{1}C_{I})}{\alpha_{2}+\alpha_{3}C_{E}+\alpha_{4}C_{I}+\alpha_{5}C_{E}C_{I}}\;,\;r=1,$$

where *T*_0_ represents transporter protein concentration on the cell membrane and *α*_1_, *α*_2_, *α*_3_, *α*_4_ and *α*_5_ represent algebraic expressions dependent on kinetic rates in the carrier model such as binding rates (see electronic supplementary material for more information).

The barrier effect provided by the carrier-mediated transport of drugs across the cell membrane allows for a discontinuity in the steady-state profile of the drug concentration distribution when there is a constant external supply that diffuses towards a metabolically active cell (as before with the passive diffusion case with low permeability). Indeed the carrier-mediated transport condition can be reduced to the passive diffusion condition mathematically with appropriate parametrization (e.g. *T*_0_ = *Q*, *α*_1,2_ = 1, *α*_3,4,5_ = 0). Furthermore, the flexibility of the carrier-mediated condition facilitates the implementation of implicit active processes whereby the flux of drug can move uphill against its concentration gradient (e.g. figure 1*c*). This can be achieved with appropriate parametrization of the simple carrier model such that *α*_1_ < 1, e.g. when binding affinity/dissociation in the interior is lower/higher than exterior binding/dissociation.

### 2.2. Parametrization

For the full multiscale model, describing the transport and metabolism of drugs in a multicellular *in vitro* environment, it is useful to include quantitative, dimensional parameter values based on experimental data to directly represent the laboratory scenario for drugs with a range of physico-chemical properties. Therefore, it is important to identify relevant parameter ranges for the microscale model before upscaling the problem to the multicellular/tissue level by introducing hepatic spheroid geometry. There are currently three key processes that determine drug dynamics in our system and require parametrization: diffusion, metabolism and permeation. For simplicity and more general applicability, we will focus on the passive diffusion case and not cover carrier-mediated transport during analysis of the multicellular model.

#### 2.2.1. Diffusion of small-molecule drugs

Most drugs, and nearly all drugs that cross the cell membrane via passive diffusion, are categorized as small-molecule drugs. These are low molecular weight (MW) compounds and comprise most drugs on the market today [21]. For a sample database of 321 such drugs [22], we calculated diffusion coefficients based on physical measurements of weight and density (MW approx. 100–1200 Da; density approx. 0.6–2.6 g m^−3^). Thus, we propose the feasible diffusion coefficient range of approximately 5 × 10^−10^ to 1 × 10^−9^ m^2^ s^−1^ (further information in the electronic supplementary material). This narrow range supports the assertion that the main determinants of drug disposition are the ability to translocate across hydrophobic diffusion barriers (permeability) and chemical transformation (metabolism), while variations in the aqueous diffusion rate have only minor effects on overall pharmacokinetics [13]. A representative value of 7.5 × 10^−10^ m^2^ s^−1^ for both *D*_I_ and *D*_E_ will be considered as default for further simulations.

#### 2.2.2. Permeability as a function of lipophilicity

The permeability of a drug transported via passive diffusion is related to its lipophilicity, a measurable physico-chemical property that can be used to define our permeability coefficient, *Q*. Ménochet *et al*. [23,24] discovered a log-linear relationship for hepatic uptake between passive diffusion clearance, *P*_diff_, and lipophilicity

2.10 $$\log P_{\mathrm{diff}}=0.6316\times \log D_{7.4}-0.3143,$$

where *P*_diff_ has units of μl min^−1^ 10^−6^ cells and log*D*_7.4_ is a partition coefficient measure of lipophilicity at a physiologically relevant pH (pH 7.4). This relationship allowed us to derive, *Q*, as a function of *P*_diff_, and the radius of the cell, *R*, by taking into account passive uptake across the whole-cell membrane of surface area 4*πR*^2^:

2.11 $$Q=\frac{P_{\mathrm{diff}}}{4\pi R^{2}}=\frac{1}{10^{6}}\frac{10^{\left(0.6316\;\times \;\log D_{7.4}-0.3143\right)}}{4\pi R^{2}}.$$

For the full derivation, see the supplementary material. log*D*_7.4_ values between 1 and 5 are considered within this study to represent relatively lipophilic, small-molecule drugs (relevant for passive diffusion), with log*D*_7.4_ = 3 as default.

#### 2.2.2. Simplified drug metabolism in hepatocytes

Metabolism represents the principal sink/removal term in our model and the metabolic rate is likely to vary greatly depending on the chemical makeup of the drug of study, as well as the quantity and activity of metabolizing enzymes present. Therefore, this term is likely to have a significant impact on the overall disposition of drug concentration in a metabolically active *in vitro* spheroid system. Metabolic rates are assumed to be independent of space in the model for simplicity, although zonal variation may exist. Brown *et al*. [25] reported kinetic parameters for a range of compounds to predict metabolic clearance by using cryopreserved human hepatocytes. This publication provided pharmacologically feasible *V*_max_ (5 × 10^−6^ to 4.5 × 10^−1^ mol m^−3^ s^−1^) and *K*_m_ (5 × 10^−4^ to 1.4 × 10^−1^ mol m^−3^) ranges for drugs primarily metabolized in the liver and were thus used as conservative guidance for this model parametrization, given that cells cultured in three-dimensional often display improved drug metabolism functions. As default, we consider parameter values of *V*_max_ = 5 × 10^−3^ mol m^−3^ s^−1^ and *K*_m_ = 1×10^−2^ mol m^−3^.

### 2.3. Macroscale: hepatocyte spheroid geometry

The impact of the hepatic spheroid environment on drug transport is considered by upscaling our microscale model to consider multiple discrete cells in a realistic spheroid geometry within an extracellular space (culture medium). This hepatocyte spheroid geometry was generated based upon histological staining of hepatic spheroids to provide representative cell sizes, number, and arrangement thereby replicating the *in vitro* scenario within the multiscale mathematical model.

#### 2.3.1. Mathematical description of spheroid geometry

**Figure 2.**
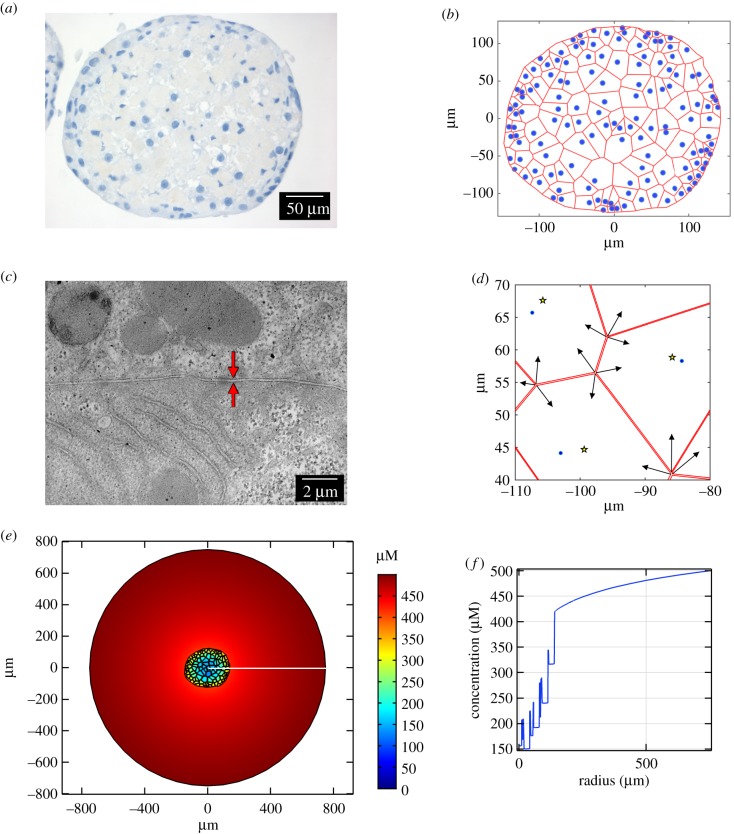
The multiscale model including hepatocyte geometry. (*a*) Histological staining of a hepatocyte spheroid slice indicating the location of cell nuclei (blue). (*b*) Voronoi diagram constructed to provide estimates of hepatocyte boundaries (red) based on the location of hepatocyte nuclei (blue). (*c*) Representative TEM image of a hepatocyte spheroid showing the size of the space between adjacent cells. The intercellular space is indicated by the red arrows. (*d*) Intercellular space was introduced into the model geometry by contracting the vertices of Voronoi cells (indicated by black arrows) towards the centre of each cell (yellow stars; nuclei in blue). (*e*) Steady-state distribution of an example drug (log*D*_7.4_ = 3 with default parameters and wide intercellular space), formed with a constant supply of 500 µM at the outer media boundary (disc of radius 750 µm). The drug distribution is denoted by the colour bar, demonstrating that there are lower drug concentrations in the central hepatocytes. (*f*) A one-dimensional cross-section of the simulation (position indicated by the white line in (*e*)) signifies the variation of drug concentration inside and outside of the cells within the spheroid structure, as well as the heterogeneity of intracellular drug concentration in different regions of the spheroid.

Histological staining of a hepatocyte spheroid revealed the spatial distribution of the cell nuclei within a section (figure 2*a*). This spatial information, as well as the spheroid boundary, was quantified digitally with WebPlotDigitizer [26] and imported into Matlab. Owing to the abundant expression of extracellular matrix in the hepatic spheroid histological images, it was not possible to visualize and/or quantify the location of the hepatocyte membranes. Therefore, we estimated the location of cell boundaries using Voronoi tessellation (figure 2*b*). Briefly, Voronoi tessellation involves assigning regions to each nucleus such that any point in space within that region is closer to that nucleus than any other. The boundaries of these regions can be determined by drawing perpendicular bisectors between adjacent pairs of nuclei. This technique has been shown to provide viable estimates for the qualitative morphology of cells in a tissue [27].

Cellular ultrastructure was visualized by transmission electron microscopy (TEM). TEM revealed that the space between hepatocytes was narrow (approx. 0.1–0.5 µm, figure 2*c*). These values are supported by the literature which states intercellular spaces from 100 nm to the µm scale [28,29]. Furthermore, it should be noted that fixation methods can shrink such morphological features [30] and therefore we consider both narrow and wide intercellular space geometries. This was achieved by contracting the vertices of each model cell towards the cell's respective centre of mass by 1% (‘narrow’, approx. 0.2 µm) or 10% (‘wide’, approx. 2 µm) (figure 2*d*).

#### 2.3.2. Experimental methods

Primary rat hepatocyte spheroids with an initial seeding density of 5000 cells were produced using the liquid-overlay technique as described by Kyffin *et al*. [31]. After 11 days in culture, the spheroids were washed in phosphate-buffered saline, fixed in 4% paraformaldehyde and subjected to routine histological processing before staining with haematoxylin or processed for TEM analysis. For TEM imaging, spheroids were fixed in 3% glutaraldehyde and processed as previously described [31]. Ultrathin (approx. 70–90 nm) sections were examined using an FEI Tecnai Transmission Electron Microscope at an accelerating voltage of 80 kV and images taken using a Gatan digital camera.

#### 2.3.3. Numerical simulation

The finite-element simulation software, COMSOL Multiphysics® 5.3, was used to solve the multiscale model PDEs. The two-dimensional spheroid slice geometry was imported into COMSOL and the PDEs were defined as before to calculate the dynamics of drug concentration, *C*, for two separate phases (intracellular, *C*_I_, and extracellular, *C*_E_):

2.12 $$\frac{\partial C_{I}}{\partial t}=D_{I}\nabla^{2}C_{I}-\frac{V_{\max}C_{I}}{C_{I}+K_{m}},\;C=C_{I}\;$$

and

2.13 $$\frac{\partial C_{E}}{\partial t}=D_{E}\nabla^{2}C_{E},\;C=C_{E},$$

with boundary conditions at every cell membrane within the spheroid,

2.14 $$(D_{I}\nabla C_{I})\cdot n=(D_{E}\nabla C_{E})\cdot n=Q(C_{E}-C_{I}),$$

for the general inward fluxes, where **n** is the unit normal vector pointing out of each cell. An illustrative example of the multiscale model steady-state with a constant supply of drug at the outer boundary of the media phase $(C_{r_{\max}}=500\;\mu M)\;$ can be seen in figure 2*e*, simulated for a drug with physico-chemical properties based on the default parameter set described above. Note that permeability *Q* is related to log*D*_7.4_ according to equation (2.11). A one-dimensional cross-section is plotted in figure 2*f* for visualization, highlighting the discontinuities in drug concentration between intra- and intercellular space and the heterogeneity in drug concentration between cells in different regions.

## 3. Results

### 3.1. Impact of drug permeability on spatio-temporal distribution throughout spheroid

**Figure 3.**
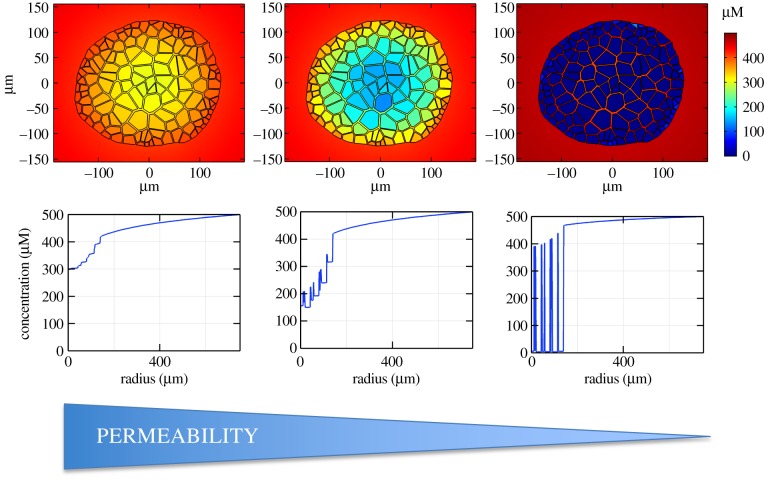
Impact of permeability on drug distribution. Top row: spatial distribution of drug concentration within a hepatic spheroid for three different permeability coefficients (permeability decreases from left to right, log*D*_7.4_ = 5, 3, 1). The plots represent steady-state values after constant supply of 500 µM at the outer media boundary (disc of radius 750 µm). Bottom row: corresponding representative one-dimensional plots are provided for each drug using the same cross-section position as figure 2*e*.

The diffusion rate of a drug depends mainly on size, a property that has minimal variation in small-molecule drugs (a detail supported by our analysis of over 300 compounds during parametrization) and thus has relatively little impact upon drug distribution when compared with the ability to translocate across the hydrophobic diffusion barrier of the cell membrane [13]. This translocation ability is determined by the lipophilicity of the drug during passive diffusional transfer across the membrane. Therefore, we consider the impact that drug permeability (as determined by lipophilicity) has upon the overall dynamics within the representative *in vitro* spheroid system. This analysis is illustrated by simulating the model, dosed for three example drugs with different permeability coefficients (corresponding to log*D*_7.4_ = 1, 3, 5, within the otherwise default parameter set) via constant supply at the external boundary and comparing the steady-state spatial distribution of drug concentration (figure 3). Spatio-temporal dynamics can be found in supplementary animations.

The results indicate that for highly lipophilic drugs (log*D*_7.4_ = 5), the cell membrane does not represent a significant barrier to drug penetration and there is relatively little difference between drug concentrations in cells and the intercellular space. For relatively lowly lipophilic drugs (log*D*_7.4_ = 1), the membranes represent a significant barrier. Drug concentration is very low within the cells but relatively high in the intercellular space throughout the spheroid. However, in the intermediate case (log*D*_7.4_ = 3), there is a relatively little drug in the spheroid centre, both inside and outside of the hepatocytes. This is due to the balance between the overall processes of drug transport towards the spheroid centre (diffusion, permeability and metabolism), impacting penetration potential. Overall, it is clear that an increase in permeability results in higher intracellular drug concentration but there is a nonlinear response in the intercellular space as permeability is increased, with a potential local minimum for drugs of intermediate lipophilicity. The same observations are made for narrow intercellular spaces and when varying transporter expression in the carrier-mediated transport model (data not shown). This result highlights the potential importance of not only permeability but also intercellular space on overall drug delivery.

### 3.2. Impact of intercellular dimensions on spatio-temporal distribution throughout spheroid

**Figure 4.**
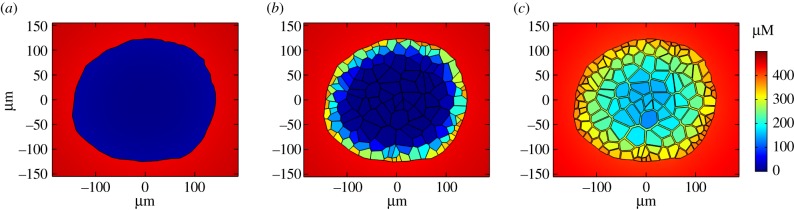
Impact of intercellular space on drug distribution. Spatial distribution of drug concentration within a hepatic spheroid for three different intercellular space geometries (no spaces (zero porosity, *a*); narrow spaces (approx. 0.2 µm, *b*); wide spaces (approx. 2 µm, *c*)). The figures represent steady-state values after constant supply of 500 µM at the outer media boundary (disc of radius 750 µm) with default parameters.

Many mathematical models of cellular spheroids consider geometrical simplifications such as radial symmetry and a homogeneous continuum of cells. The consideration of a spheroid with individual hepatocytes modelled as discrete regions in space, and accompanying intercellular space, has a visible impact upon the radial drug concentration profile. This can be seen most clearly in the case of low permeability with large fluctuations in the drug concentration between intra- and intercellular space (figure 3). There is a considerable range of intercellular gap sizes within spheroids, a feature which can be magnified by fixation issues and cell type, with tumour spheroids notoriously exhibiting higher porosities [32]. Therefore, it is prudent to also consider the impact of porosity (gap size) on drug delivery by simulating our model for both narrow and wide intercellular space geometries, as well as a model without intercellular space altogether for comparison. Steady-state spatial distributions in figure 4 suggest that intercellular space has a considerable impact on drug penetration, with increased porosity resulting in higher drug concentration for the spheroid interior.

### 3.3. Translating the multiscale model to a simple continuum model

From figure 4 it is clear that, when using quantitative, measurable, microscale parameters, the assumption of a homogeneous continuum of hepatocytes in the spheroid will be insufficient for simulating spatial drug distributions, particularly for wider intercellular spaces. Therefore, we consider if there are any parameter modifications that can be made such that the continuum model can be said to sufficiently replicate the simulations provided by the more spatially complex discretized model. Such a model would be highly beneficial for the quantification of drug dynamics with greater computational efficiency. For this investigation, we compare the average behaviour of the full discrete, multiscale, dimensional model (cell-based model) with the idealized radially symmetric, homogenized sphere model (continuum model) in two-dimensional (cylindrical coordinates) given by

3.1 $$\frac{\partial C_{S}}{\partial t}=\frac{D_{I}^{\mathrm{Eff}}}{r}\frac{\partial }{\partial r}\left(r\frac{\partial C_{S}}{\partial r}\right)-\frac{V_{\max}C_{S}}{C_{S}+K_{m}},\;r\leq R_{S}\;$$

and

3.2 $$\frac{\partial C_{O}}{\partial t}=\frac{D_{E}}{r}\frac{\partial }{\partial r}\left(r\frac{\partial C_{O}}{\partial r}\right),\;r>R_{S},$$

where *C*_S_ and *C*_O_ represent spheroid and outer drug concentrations, respectively, and *R*_S_ = 135 µm (the average radius of the hepatocyte sphere slice in figure 2), with boundary conditions

3.3 $$D_{I}^{\mathrm{Eff}}\frac{\partial C_{S}}{\partial r}=0,\;r=0\;$$

and

3.4 $$D_{I}^{\mathrm{Eff}}\frac{\partial C_{S}}{\partial r}=D_{E}\frac{\partial C_{O}}{\partial r}\;=Q^{\mathrm{Eff}}(C_{O}-C_{S}),\;r=R_{S},$$

for effective parameters $D_{I}^{\mathrm{Eff}}$ and *Q*^Eff^ which represent the parameters to be modified. These parameters are logical targets for the translation since they determine interior transport via internal diffusion and translocation across cell membranes in the cell-based model. Homogenization here can be thought of as an extreme modification of the spheroid structure such that we reduce the system to a very large single cell with a single permeable membrane. The effective parameter values of the continuum model were optimized to fit the average behaviour of the cell-based models for both intercellular space geometries and a physico-chemically relevant range of permeability coefficients (corresponding to log*D*_7.4_ = 1, 2, 3, 4, 5). For information regarding parameter optimization, see the supplementary material.

**Figure 5.**
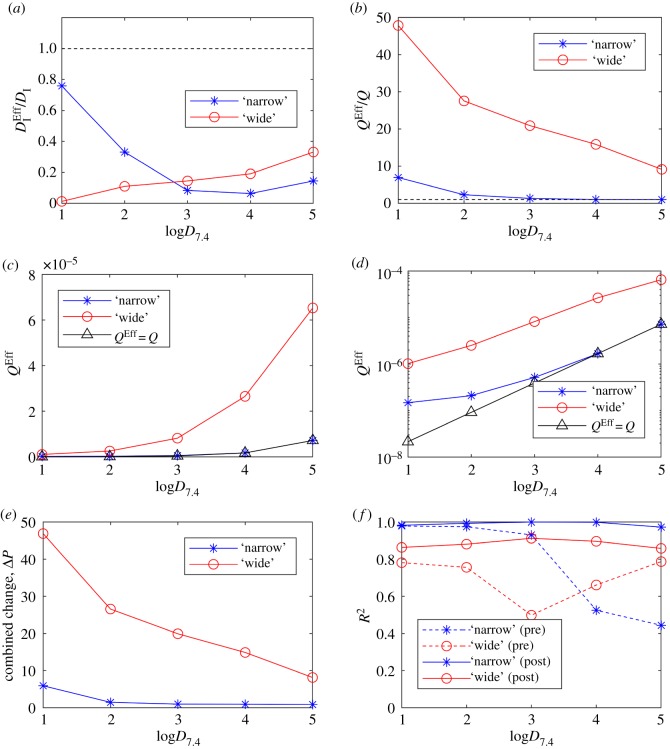
Emulating the cell-based models with a simple, symmetric continuum model. Effective parameters for intracellular diffusion and permeability ($D_{I}^{\mathrm{Eff}}$ and *Q*^Eff^) were optimized in the continuum model to fit the output for the cell-based models for a range of drug lipophilicities. (*a*) The optimized $D_{I}^{\mathrm{Eff}}$ value provides the required relative changes in intracellular diffusion for the continuum model to match the cell-based models with narrow (blue) and wide (red) intercellular space geometries. (*b*) Required relative changes in permeability identified by optimizing *Q*^Eff^. Absolute values of *Q*^Eff^ optimized for each cell-based model and drug lipophilicity (log*D*_7.4_) are plotted in (*c*). (*d*) Optimized *Q*^Eff^ on a log scale (*y*-axis). (*e*) A combined parameter change metric (Δ*P*) indicates the summarized amount of model modification required for the continuum model to effectively simulate the cell-based models. (*f*) Comparisons between model outputs are calculated by using the *R*^2^ error metric to determine relative quality of fits (see electronic supplementary material for definition). Comparisons are made both prior to optimization (‘pre’, direct comparison using dimensional parameters, i.e. $D_{I}^{\mathrm{Eff}}=D_{I},\;Q^{\mathrm{Eff}}=Q$) and post-optimization (post).

The required modifications of effective parameters, both collectively and individually as functions of drug lipophilicity and intercellular space, are summarized in figure 5, as well as corresponding error metrics. A combined parameter change metric in figure 5*e* is introduced to quantify the relative amount of modification required for each scenario (intercellular width and lipophilicity) and defined as

3.5 $$\Delta P=\sqrt{{\left(\frac{D_{I}^{\mathrm{Eff}}-D_{I}}{D_{I}}\right)}^{2}+{\left(\frac{Q^{\mathrm{Eff}}-Q}{Q}\right)}^{2}}.$$

From figure 5, it is clear that Δ*P* is dominated by relative changes in the effective permeability coefficient, *Q^Eff^* (compare figure 5*e* with figure 5*a*,*b*). Permeability must be increased to account for the intercellular space in the cell-based models (all lipophilicities), i.e. *Q*^Eff^/*Q* ≥ 1 for all log*D*_7.4_ (figure 5*b*). This effectively makes the spheroid boundary in the continuum model more porous (virtually simulating gaps between cells) and the discontinuity at the spheroid boundary is reduced. It should be noted that in the dimensional cell-based models, while *D*_I_ remains constant throughout all simulations, *Q* will change dependent on log*D*_7.4_ (recall equations (2.10)–(2.11)). This is seen in figure 5*c*,*d* with absolute changes in *Q*^Eff^ and *Q*. Permeability must be increased by a greater amount for wider intercellular spaces to be effectively simulated by the continuum model (e.g. figure 5*d*) for all log*D*_7.4_. This is expected due to the increased porosity provided by wider gaps. Finally, effective permeability must be increased by a greater amount for low lipophilicities. This can be seen in figure 5*b* where the effective permeability *Q*^Eff^ decreases towards the dimensional value *Q* with increasing lipophilicity, for both gap sizes, in a monotonic fashion. This reflects the increased discrepancy between transport through cells and transport between cells found for drugs that are poorly lipid soluble.

In order for the continuum model to effectively simulate intercellular space, intracellular diffusion must be decreased for all lipophilicities, i.e. $D_{I}^{\mathrm{Eff}}<D_{I}$ for all log*D*_7.4_ (figure 5*a*). The primary effect of decreasing this parameter in the model is to increase the gradient of concentrations within the spheroid. For high lipophilicity and narrow intercellular spaces, the continuum model can provide a representative simulation of the cell-based model by reducing $D_{I}^{\mathrm{Eff}}$ alone. This property is observed by comparing the negligible changes in *Q*^Eff^ relative to $D_{I}^{\mathrm{Eff}}$ at high lipophilicity and narrow intercellular spaces. For example, when log*D*_7.4_ = 4 and 5, *D*_I_ is decreased by 94 and 86%, while *Q* is unchanged (figure 5*a*,*b*). Theoretically, given a high enough value of log*D*_7.4_, this behaviour is expected for wide spaces too, but this is beyond relevant parameter space.

Regardless of lipophilicity, the optimized continuum model compares better with the cell-based model of narrow intercellular gaps (figure 5*f*, solid lines). This is likely due to the relatively lower amount of fluctuations in the mean one-dimensional profiles as there is less extracellular space in general within the spheroid. These fluctuations represent the local drug concentration variation at the cellular scale due to discrepancies between intra- and extracellular phases, which can be very high for drugs that are poorly lipid soluble (e.g. figure 3 one-dimensional profiles). Prior to any optimization and rescaling of dimensional parameters to their effective counterparts $\left(D_{I}\to D_{I}^{\mathrm{Eff}},Q\to Q^{\mathrm{Eff}}\right)$, there was a clear pattern in the fit quality between the simple continuum model approximation and the cell-based models of different sized intercellular spaces (figure 5*f*, dashed lines). Generally, the continuum model fits the narrow spaces better for low membrane permeability and wider spaces better for high permeability. This feature appears to be correlated to the overall higher intracellular drug concentrations found in spheroids with wider spaces (since there is proportionally less transport across metabolizing cells). The (pre-optimized) continuum model exhibits very low drug concentration within the spheroid at low permeability and so fits the narrow-spaced model better. At higher permeabilities, the continuum model has relatively high interior concentration and so fits the wide spaced cell-based model better (electronic supplementary material, figure S1). This switch in behaviour is likely due to the continuum model only providing a single barrier to permeation (spheroid boundary), which, once penetrated, facilitates drug penetration via diffusion solely.

Interestingly, figure 5*e* indicates that the cell-based model with wider intercellular spaces requires more parameter modification for all drug lipophilicities. Despite the intra-spheroidal gradients being vastly different between the (pre-optimized) continuum and narrow cell-based model at high permeabilities (electronic supplementary material, figure S1), the boundary intracellular drug concentrations are similar. Therefore, the continuum model can be optimized via sufficient reduction in $D_{I}^{\mathrm{Eff}}$ while maintaining the original permeability coefficient (*Q*^Eff^ = *Q*). However, in order to simulate the wide cell-based model, and account for different concentrations in boundary cells, a relatively greater change in *Q*^Eff^ was required (compare relative changes in effective parameters at log*D*_7.4_ = 4 and 5 for $D_{I}^{\mathrm{Eff}}$ and *Q*^Eff^ for both models, figure 5*a*,*b*).

### 3.4. Investigating the impact of permeability on the dynamic process of drug delivery in different regions of the spheroid for a bolus dose

Intercellular space has a discernible impact on the spatio-temporal drug dynamics in the *in vitro* spheroid environment and moreover, a nonlinear effect was revealed for local concentrations within intercellular space as permeability is increased (figure 3). Since this phenomenon (i.e. a monotonic decrease in intracellular drug concentration with decreasing permeability, but a non-monotonic response in the intercellular regions) cannot be described by the simple continuum model, it is worth considering the potential impact of this feature on drug penetration. Here, we choose to examine drug delivery and subsequent effects by calculating the total uptake/metabolism of the drug in different regions of the spheroid. To investigate drug delivery via metabolism, we introduce the following ‘metabolism’ variable, *M*, with dynamics

3.6 $$\frac{\partial M}{\partial t}=\frac{V_{\max}C}{C+K_{m}},\;C=C_{I},$$

which corresponds to accumulated drug metabolized and is only relevant inside model cells. Corresponding model simulations are conducted with a finite bolus dose initially supplied in the outer medium, uniformly distributed in the extracellular space outside the spheroid, and zero-flux boundary conditions are imposed on the outer boundary of the media phase.

**Figure 6.**
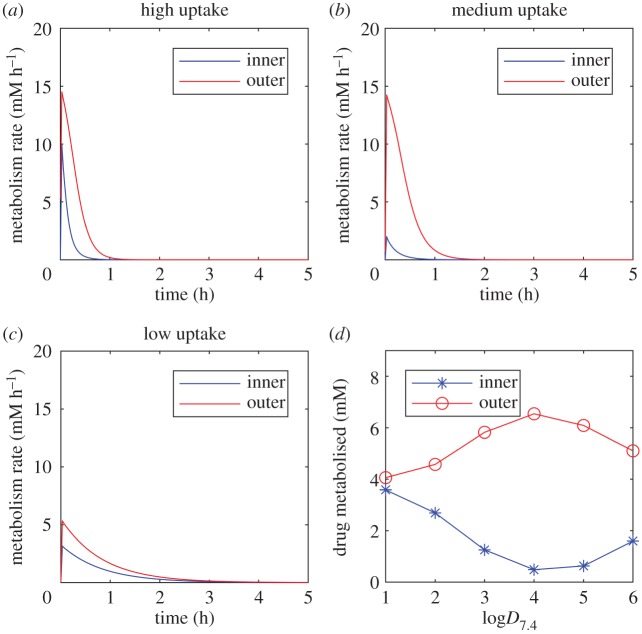
Impact of drug lipophilicity on uptake and metabolism in different regions of the spheroid. Metabolism rates are plotted against time as a result of model simulations following a bolus dose of 100 µM, initially uniformly distributed in the medium for varying drug lipophilicities (log*D*_7.4_ = 6 (*a*), 4 (*b*), 2 (*c*)). (*d*) Total metabolized drug (after complete clearance) is compared for inner and outer hepatocytes within the spheroid for a range of drug lipophilicities (log*D*_7.4_ = 1–6).

Two separate regions are defined, ‘outer’ and ‘inner’, corresponding to cells of comparable size in the outer boundary layer of the spheroid, (*x*, *y*) = (−10 µm, 110 µm), and the spheroid centre, (*x*, *y*) = (0 µm, 0 µm). Simulations are run to the drug-free steady-state whereby all of the initial dose has been removed from the system and accumulated in the effective sink variable, *M*. For highly lipophilic drugs, the concentration dynamics are relatively similar between inner and outer regions as the drug is able to be transported throughout the spheroid quickly, unrestricted by permeability. However, the outer cells are exposed to slightly higher concentrations and consequently more drug is metabolized in this region, demonstrated by similar rates of metabolism (figure 6*a*). Simulations of lowly lipophilic drugs require much longer timespans in order to reach equilibrium due to the reduced uptake rate at the cell membranes. However, due to the intercellular transport via diffusion, even centrally located cells receive relatively high local drug exposure and metabolize at a similar rate to outer cells (figure 6*c*). It is the *in silico* drugs of intermediate lipophilicity in this model scenario that exhibits the most striking discrepancies between inner and outer cells (figure 6*b*). The impact of these varying rates of metabolism between drug lipophilicities and regions of the spheroid can be evaluated by comparing the total drug metabolized (figure 6*d*). The greatest discrepancy in drug uptake between outer and inner hepatocytes is revealed for drugs of intermediate permeability (1250% increase from inner to outer cells for log*D*_7.4_ = 4 compared with just +13% for log*D*_7.4_ = 1 and +219% for log*D*_7.4_ = 6). Furthermore, outer cells in this case receive the most drug out of all three cases studies and the inner cells receive the least (figure 6*d*). This effect can potentially be exacerbated when carrier-mediated transport kinetics are modelled at the cell membrane, due to the saturating effects of this uptake mechanism (arbitrary transporter parametrization, data not shown). This feature has the potential to significantly impact experimental design considerations and *in vitro* drug efficacy and toxicity evaluation.

## 4. Discussion

The enhanced sophistication of current cell culture methodologies due to increasing advancements in scientific understanding and technological developments has allowed for *in vitro* studies to become more physiologically relevant. There is a range of different *in vitro* models that span varying levels of complexity, reproducibility, high-throughput potential and cost. Spheroids represent an intermediate experimental model that allows for increased physiological relevance over two-dimensional monolayers due to the three-dimensional environment, as well as more appropriate cell morphology and functionality while remaining cost-effective, consistent and easy to use [1]. The subsequent prevalence of liver spheroid cultures for studying hepatocyte behaviour *in vitro* is evident and represents a key component of drug development such that drug candidates can be tested for efficacy and toxic potential in a three-dimensional environment with physiological gradients [31,33–35]. Data-driven multiscale mathematical models provide an ideal platform from which to try and enhance mechanistic understanding of new biotechnologies by simulating the underlying physical processes. Additionally, the development of spatio-temporal data generated by three-dimensional cell imaging offers tremendous opportunities for developing, parametrizing and testing multiscale mathematical models and in response, mathematical modelling can be successfully used to optimize these developing technologies.

In this study, we developed a mathematical model of drug transport and metabolism in a multiscale spheroid framework, accounting for microscale processes such as membrane transport kinetics and how they relate to the physico-chemical properties of a drug, and macroscale features such as the geometry of a hepatocyte spheroid, informed by imaging data. Cellular uptake of drugs was modelled by the two major processes of transport across the cell membrane, passive diffusion and carrier-mediated transport [17]. The carrier-mediated transport microscale model was innately more complex, depending on quantities such as transporter protein expression, binding kinetics and rates of conformational change and this complexity allowed for a wider array of dynamic mechanisms such as enzymatic saturation and active processes. The extensive parametrization required to quantify the carrier-mediated transport model depends on more compound-specific information, and so the passive diffusion case became the main focus of investigations within the generalized multiscale framework, more relevant for relatively lipophilic compounds.

The explicit representation of individual hepatocytes based on imaging data allowed for an investigation into the effects of including a distinct cell-based geometry in the model. The model suggests that steady-state intracellular drug concentrations increase monotonically with increasing drug lipophilicity. However, a non-monotonic relationship was revealed between drug lipophilicity and intercellular drug concentration (figure 3), while the width of the intercellular space further impacted spatial drug distribution (figure 4). Intercellular space geometry, or spheroid porosity, is therefore a key physiological feature of the multicellular structure but is both difficult to accurately quantify and known to vary widely between cell types. This is particularly relevant in the case of tumour-derived spheroids, whose morphology tends to be more porous [32], and organoids that are increasingly being used in efficacy testing for tumour cells [36]. We, therefore, studied two different average intercellular widths informed by TEM data and the literature which suggested a range of 10^2^–10^3^ nm scale, with results varying due to cell type, tumour phenotype and experimental artefacts such as fixation [29,30,37].

While it is important to account for intercellular space within spheroids to correctly model drug delivery, the consequent increase in complexity by modelling this feature explicitly renders detailed analytic work intractable and deriving numerical solutions is costly with respect to time and computational power requirements. Therefore, it is appropriate to consider the application of simplified models that consider averaged or homogenized system behaviour and under what conditions they can provide valid approximations [38]. We have shown how to approximate the cell-based models using a simple, symmetric, continuum model by reparametrizing dimensional parameters to re-scaled effective counterparts. For relatively narrow intercellular gaps, these approximations are more accurate and the required parameter changes are reduced. The differences between the models, due to the explicit representation of intercellular space (porosity) within the cell-based model, are largely accounted for by increasing the effective permeability parameter. This increase in the effective permeability increases the drug transport across the spheroid boundary in the continuum model. This is particularly important at lower lipophilicities when permeability limitations are maximized. For higher lipophilicities and narrow intercellular space, the spatially averaged dynamics of the cell-based model can be effectively simulated with the symmetric continuum model by appropriate reductions in the effective internal diffusion parameter only. Further work is required to determine the impact of spatially varying quantities that might exist within a spheroid such as intercellular space or transporters that vary zonally [39], and how these might compare between continuum models and cell-based models. Metabolic rates are also known to vary in space throughout multicellular structures due to gradients in environmental factors such as oxygen and glucose [40]. Alternative model simplifications that might expedite analysis can be made by careful consideration of potentially redundant model complexities such as intracellular diffusion, which may be neglected in some scenarios. The model currently neglects any intracellular binding of the drug for simplicity, focusing on the dominant mechanisms of transport and removal (metabolism) that drive the spatio-temporal dynamics. However, for specific future applications of the model, intracellular binding could be considered by ascertaining the relevant fraction unbound for a particular drug, as this will lower the rate of metabolism for those drugs which bind strongly to intracellular proteins and nuclear structures.

The discovery of an apparent local minimum in drug penetration, whereby intercellular concentrations are lower for intermediate membrane permeation, motivated an investigation into corresponding effects on drug delivery, uptake and metabolism in spheroid centres for a bolus dose (figure 6). The results of this investigation indicated that, indeed, it is feasible to observe minimal drug uptake at the spheroid centre for drugs of intermediate lipophilic properties (with the majority of the drug being metabolized at the outer regions). These mechanistic insights and modelling results have potential impact for the dosing of spheroid systems *in vitro* as well as relevance for analogous *in vivo* systems such as avascular tumours. It is not necessarily sufficient to assume that increasing a chemical's lipid solubility will enhance its metabolism at the spheroid centre. Lowly lipid soluble drugs may require a much longer time in culture but ultimately metabolize the drug more uniformly throughout the spheroid. Accounting for reduced penetration due to the intermediate lipophilic property may be alleviated somewhat by increasing the dose, but this could have potentially toxic consequences from overdosing cells at the spheroid boundary. Other experimental design options include manipulating permeability (by chemical modification or intervention, but this could potentially further increase the divergent amounts of drug being metabolized in different regions of the spheroid) or using smaller spheroids. These investigations could be conducted within the *in silico* framework, in the first instance, to guide strategy. The implications of drug delivery characteristics based on permeability parameters could potentially be translated to targeting delivery in tissues of multiple cell types expressed zonally. For example, targeting the central zone of a spheroid that contains cells of a different phenotype (e.g. cancerous/hypoxic) may be aided by manipulating these properties regarding permeability, i.e. making certain that the permeability is either relatively high or relatively low to ensure delivery to the spheroid centre. Validation of these *in silico* investigations could involve emerging technologies such as MALDI (matrix-assisted laser desorption/ionization)-mass spectrometry imaging, which provide label-free mass spectrometric detection within tissue sections [41]. This detection methodology is rapidly being developed to provide a quantitative measure of drug penetration within a tissue/spheroid at different time-points that could potentially be compared with our model. The combination of mathematical modelling with experimental imaging provides a convenient *in silico* testing toolkit to optimize the use of three-dimensional cell culture systems in the laboratory and maximize the potential of spheroid models aiding drug discovery, toxicity testing and dose optimization.

## Supplementary Material

Supplementary material

## Supplementary Material

Supplementary Animations

## Data accessibility

This article has no additional data.

## References

[1] Kyffin JA, Sharma P, Leedale J, Colley HE, Murdoch C, Mistry P, Webb SD 2018 Impact of cell types and culture methods on the functionality of *in vitro* liver systems-a review of cell systems for hepatotoxicity assessment. Toxicol. In Vitro 48, 262–275. (10.1016/j.tiv.2018.01.023) 29408671

[2] Hoarau-Véchot J, Rafii A, Touboul C, Pasquier J 2018 Halfway between 2D and animal models: are 3D cultures the ideal tool to study cancer-microenvironment interactions? Int. J. Mol. Sci. 19, 181 (10.3390/ijms19010181) PMC5796130 29346265

[3] Fang Y, Eglen RM 2017 Three-dimensional cell cultures in drug discovery and development. SLAS Discov. 22, 456–472. (10.1177/1087057117696795) 28520521 PMC5448717

[4] Bell CC et al. 2018 Comparison of hepatic 2D sandwich cultures and 3D spheroids for long-term toxicity applications: a multicenter study. Toxicol. Sci. 162, 655–666. (10.1093/toxsci/kfx289) 29329425 PMC5888952

[5] Williams DP, Shipley R, Ellis MJ, Webb S, Ward J, Gardner I, Creton S 2013 Novel *in vitro* and mathematical models for the prediction of chemical toxicity. Toxicol. Res. 2, 40–59. (10.1039/C2TX20031G) PMC4765367 26966512

[6] Karolak A, Markov DA, McCawley LJ, Rejniak KA 2018 Towards personalized computational oncology: from spatial models of tumour spheroids, to organoids, to tissues. J. R Soc. Interface 15, 20170703 (10.1098/rsif.2017.0703) 29367239 PMC5805971

[7] Visser S, Alwis D, Kerbusch T, Stone J, Allerheiligen S 2014 Implementation of quantitative and systems pharmacology in large pharma. CPT Pharmacometrics Syst. Pharmacol. 3, 1–10. (10.1038/psp.2014.40) 25338195 PMC4474169

[8] Sturla SJ, Boobis AR, FitzGerald RE, Hoeng J, Kavlock RJ, Schirmer K, Whelan M, Wilks MF, Peitsch MC 2014 Systems toxicology: from basic research to risk assessment. Chem. Res. Toxicol. 27, 314–329. (10.1021/tx400410s) 24446777 PMC3964730

[9] Turner RM, Park BK, Pirmohamed M 2015 Parsing interindividual drug variability: an emerging role for systems pharmacology. Wiley Interdiscip. Rev.: Syst. Biol. Med. 7, 221–241. (10.1002/wsbm.1302) 25950758 PMC4696409

[10] Krewski D et al 2010 Toxicity testing in the 21st century: a vision and a strategy. J. Toxicol. Environ. Health B Crit Rev 13, 51–138. (10.1080/10937404.2010.483176) 20574894 PMC4410863

[11] Raies AB, Bajic VB 2016 In silico toxicology: computational methods for the prediction of chemical toxicity. Wiley Interdiscip. Rev.: Comput. Mol. Sci. 6, 147–172. (10.1002/wcms.1240) 27066112 PMC4785608

[12] Pridgeon CS et al. 2018 Innovative organotypic *in vitro* models for safety assessment: aligning with regulatory requirements and understanding models of the heart, skin, and liver as paradigms. Arch. Toxicol. 92, 557–569. (10.1007/s00204-018-2152-9) 29362863 PMC5818581

[13] Rang H, Dale M, Ritter J, Moore P 2003 Pharmacology, 5th edn New York, NY: Churchill Livingston.

[14] Dobson PD, Kell DB 2008 Carrier-mediated cellular uptake of pharmaceutical drugs: an exception or the rule? Nat. Rev. Drug Discovery. 7, 205–220. (10.1038/nrd2438) 18309312

[15] Kell DB 2015 What would be the observable consequences if phospholipid bilayer diffusion of drugs into cells is negligible? Trends Pharmacol. Sci. 36, 15–21. (10.1016/j.tips.2014.10.005) 25458537

[16] Sugano K et al. 2010 Coexistence of passive and carrier-mediated processes in drug transport. Nat. Rev. Drug Discovery. 9, 597–614. (10.1038/nrd3187) 20671764

[17] Cocucci E, Kim JY, Bai Y, Pabla N 2017 Role of passive diffusion, transporters, and membrane trafficking-mediated processes in cellular drug transport. Clin. Pharmacol. Therapeut. 101, 121–129. (10.1002/cpt.545) 27804130

[18] Nigam SK 2015 What do drug transporters really do? Nat. Rev. Drug Discovery. 14, 29 (10.1038/nrd4461) 25475361 PMC4750486

[19] Keener JP, Sneyd J 1998 Mathematical physiology. Berlin, Germany: Springer.

[20] Wood BD, Whitaker S 1998 Diffusion and reaction in biofilms. Chem. Eng. Sci. 53, 397–425. (10.1016/S0009-2509(97)00319-9)

[21] AstraZeneca. Small Molecules. 2019 See https://www.astrazeneca.com/what-science-can-do/drug-modalities/small-molecule.html.

[22] Kyffin JA 2018 Establishing species-specific 3D liver microtissues for repeat dose toxicology and advancing *in vitro* to *in vivo* translation through computational modelling. PhD Thesis, Liverpool John Moores University.

[23] Ménochet K, Kenworthy KE, Houston JB, Galetin A 2012 Use of mechanistic modeling to assess interindividual variability and interspecies differences in active uptake in human and rat hepatocytes. Drug Metab. Dispos. 40, 1744–1756. (10.1124/dmd.112.046193) 22665271 PMC3422540

[24] Ménochet K, Kenworthy KE, Houston JB, Galetin A 2012 Simultaneous assessment of uptake and metabolism in rat hepatocytes: a comprehensive mechanistic model. J. Pharmacol. Exp. Ther. 341, 2–15. (10.1124/jpet.111.187112) 22190645 PMC3310695

[25] Brown HS, Griffin M, Houston JB 2007 Evaluation of cryopreserved human hepatocytes as an alternative *in vitro* system to microsomes for the prediction of metabolic clearance. Drug Metab. Dispos. 35, 293–301. (10.1124/dmd.106.011569) 17132764

[26] Rohatgi A 2018 WebPlotDigitizer Austin, Texas, USA2018 [4.1]. See: https://automeris.io/WebPlotDigitizer.

[27] Kaliman S, Jayachandran C, Rehfeldt F, Smith A-S 2016 Limits of applicability of the Voronoi tessellation determined by centers of cell nuclei to epithelium morphology. Front. Physiol. 7, 551 (10.3389/fphys.2016.00551) 27932987 PMC5122581

[28] Goodman TT, Chen J, Matveev K, Pun SH 2008 Spatio-temporal modeling of nanoparticle delivery to multicellular tumor spheroids. Biotechnol. Bioeng. 101, 388–399. (10.1002/bit.21910) 18500767 PMC2835857

[29] Gao Y, Li M, Chen B, Shen Z, Guo P, Wientjes MG, Au JL-S 2013 Predictive models of diffusive nanoparticle transport in 3-dimensional tumor cell spheroids. AAPS J. 15, 816–831. (10.1208/s12248-013-9478-2) 23605950 PMC3691442

[30] Chatterjee S 2014 Artefacts in histopathology. J. Oral Maxillofac. Pathol. 18(Suppl 1), S111 (10.4103/0973-029X.141346) 25364159 PMC4211218

[31] Kyffin JA, Sharma P, Leedale J, Colley HE, Murdoch C, Harding AL, Mistry P, Webb SD 2019 Characterisation of a functional rat hepatocyte spheroid model. Toxicol. In Vitro 55, 160–172. (10.1016/j.tiv.2018.12.014) 30578835 PMC6361770

[32] Li Y, Wang J, Wientjes MG, Au JL-S 2012 Delivery of nanomedicines to extracellular and intracellular compartments of a solid tumor. Adv. Drug Deliv. Rev. 64, 29–39. (10.1016/j.addr.2011.04.006) 21569804 PMC3378679

[33] Andersson TB 2017 Evolution of novel 3D culture systems for studies of human liver function and assessments of the hepatotoxicity of drugs and drug candidates. Basic Clin. Pharmacol. Toxicol. 121, 234–238. (10.1111/bcpt.12804) 28470941

[34] Hendriks DF, Puigvert LF, Messner S, Mortiz W, Ingelman-Sundberg M 2016 Hepatic 3D spheroid models for the detection and study of compounds with cholestatic liability. Sci. Rep. 6, 35434 (10.1038/srep35434) 27759057 PMC5069690

[35] Bell CC et al. 2016 Characterization of primary human hepatocyte spheroids as a model system for drug-induced liver injury, liver function and disease. Sci. Rep. 6, 25187 (10.1038/srep25187) 27143246 PMC4855186

[36] Kopper O et al. 2019 An organoid platform for ovarian cancer captures intra-and interpatient heterogeneity. Nat. Med. 25, 838. 31011202 10.1038/s41591-019-0422-6

[37] Leroux C-E, Monnier S, Wang I, Cappello G, Delon A 2014 Fluorescent correlation spectroscopy measurements with adaptive optics in the intercellular space of spheroids. Biomed. Opt. Express 5, 3730–3738. (10.1364/BOE.5.003730) 25360385 PMC4206337

[38] Wood BD, Quintard M, Whitaker S 2002 Calculation of effective diffusivities for biofilms and tissues. Biotechnol. Bioeng. 77, 495–516. (10.1002/bit.10075) 11788949

[39] Tomlinson L, Hyndman L, Firman JW, Bentley R, Kyffin JA, Webb SD, Mcginty S, Sharma P 2019 *In vitro* liver zonation of primary rat hepatocytes. Front. Bioeng. Biotechnol. 7, 17 (10.3389/fbioe.2019.00017) 30834246 PMC6387900

[40] Sheth DB, Gratzl M 2019 Electrochemical mapping of oxygenation in the three-dimensional multicellular tumour hemi-spheroid. Proc. R. Soc. A 475, 20180647 (10.1098/rspa.2018.0647) 31236040 PMC6545061

[41] Winter M, Bretschneider T, Kleiner C, Ries R, Hehn JP, Redemann N, Luippold AH, Bischoff D, Büttner FH 2018 Establishing MALDI-TOF as versatile drug discovery readout to dissect the PTP1B enzymatic reaction. SLAS Discov. 23, 561–573. (10.1177/2472555218759267) 29466676

