# Supplementary material for: Multiscale modelling of drug transport and metabolism in liver spheroids
Source: Interface Focus. 2020 Feb 14;10(2):20190041. doi: 10.1098/rsfs.2019.0041 (PMC7061947; doi:10.1098/rsfs.2019.0041)
Supplement: Supplementary material [file rsfs20190041supp1.pdf]

SUPPLEMENTARY MATERIAL:

Multiscale modelling of drug transport and metabolism in liver spheroids

Joseph A. Leedale[1], Jonathan A. Kyffin[2], Amy L. Harding[3], Helen E. Colley[3], Craig Murdoch[3], Parveen Sharma[4], Dominic P. Williams[5], Steven D. Webb[1,2], Rachel N. Bearon[1]

[1]EPSRC Liverpool Centre for Mathematics in Healthcare, Dept. of Mathematical Sciences, University of Liverpool, Liverpool, L69 7ZL, UK

[2]Dept. of Applied Mathematics, Liverpool John Moores University, Liverpool, L3 3AF, UK

[3]School of Clinical Dentistry, University of Sheffield, Claremont Crescent, Sheffield, S10 2TA, UK

[4]MRC Centre for Drug Safety Science, Dept. of Molecular and Clinical Pharmacology, University of Liverpool, Liverpool, L69 3GE, UK

[5]AstraZeneca, IMED Biotech Unit, Drug safety & Metabolism, Cambridge Science Park, Cambridge, CB4 0FZ, UK

Contents

1 Microscale Transport 3

1.1 Passive diffusion 4

1.1.1 Numerical solution 5

1.1.1.1 Sphere centre boundary 6

1.1.1.2 Phase interface boundary 6

1.1.1.3 External boundary 7

1.1.2 Model simulations 8

1.2 Carrier-mediated transport 8

1.2.1 Numerical solution 9

2 Parameterisation of passive diffusion 11

2.1 Diffusion of small molecule drugs 11

2.2 Permeability as a function of lipophilicity 11

3 Translating the multiscale model to a simple continuum model 13

3.1 Optimisation of effective parameters for the simple continuum model 13

References 16

1 Microscale Transport

The mathematical representation of microscale drug transport across a cell membrane can be studied with a simple model that considers the processes governing drug concentration dynamics in two phases, inside and outside the cell, with a permeable barrier in-between. Assume diffusion occurs at different rates inside (D_I) and outside (D_E) of a spherical cell of radius R and that the drug is metabolised within the cell. The drug concentration (C) dynamics inside the cell are given by the partial differential equation (PDE):

$$\frac{\partial C}{\partial \tilde{t}} = \tilde{\nabla}^2 C - \frac{\tilde{V}_{max} C}{C + K_m}, \tag{S1}$$

with scaling

$$\tilde{\boldsymbol{x}} = \frac{\boldsymbol{x}}{R}, \qquad \tilde{t} = \frac{D_I}{R^2} t, \qquad \tilde{V}_{max} = \frac{R^2}{D_I} V_{max}, \tag{S2}$$

where V_{max} is the maximum metabolism rate and K_m represents the drug concentration at which metabolism is half maximal. Note that the metabolism of the drug is assumed to occur with Michaelis-Menten kinetics, which is relevant for enzyme-mediated biochemical reactions but here prohibits the derivation of an analytical solution at the steady state. Assume that outside the cell drug transport is governed by diffusion processes only:

$$\frac{\partial C}{\partial \tilde{t}} = D \tilde{\nabla}^2 C, \tag{S3}$$

where $D = D_E / D_I$. For simplicity, assume that the problem is radially symmetric, drop the tildes and convert to spherical coordinates for a 1D representative model with respect to the radius, r.

$$\frac{\partial C}{\partial t} = \frac{1}{r^2} \frac{\partial}{\partial r}\left(r^2 \frac{\partial C}{\partial r}\right) - \frac{V_{max} C}{C + K_m}, \qquad r \leq 1, \tag{S4}$$

$$\frac{\partial C}{\partial t} = \frac{D}{r^2} \frac{\partial}{\partial r}\left(r^2 \frac{\partial C}{\partial r}\right), \qquad r > 1. \tag{S5}$$

We impose the following boundary conditions for the cell centre ($r = 0$) and a distance away from the cell $r = r_{max}$:

$$\frac{\partial C}{\partial r} = 0, \qquad r = 0, \tag{S6}$$

$$C = C_{r_{max}}, \qquad r = r_{max}, \tag{S7}$$

where $C_{r_{max}}$ is a constant supply term to be prescribed. Assume that the flux at the sphere boundary is equal such that mass is conserved, i.e.,

$$D_I \frac{\partial C_I}{\partial r} = D_E \frac{\partial C_E}{\partial r}, \qquad r = 1, \tag{S8}$$

where C_I and C_E are used to distinguish between interior and exterior drug concentrations at the cell membrane boundary. A further boundary condition must be specified at the cell membrane boundary in order to solve the coupled PDE system and investigate the effects of different means of drug transport.

1.1 Passive diffusion

To determine the boundary condition describing the flux of drug into the cell due to passive diffusion, consider an additional compartment, i.e., the cell membrane. It is assumed that within this compartment, drug transport is determined solely by aqueous diffusion. Since the thickness of the membrane (~5-10 nm) is much smaller than the cell radius (~10-20 μm) and surrounding cell space, it is assumed that the drug diffuses across the space between the lipid barriers of the membrane relatively quickly compared to transport outside. Therefore, we assume that there is a valid quasi-steady state assumption to be made at either side of the membrane such that drug concentration can be assumed to be constant at the lipid barriers on this quick timescale. Mathematically, we can represent this as a thin membrane compartment of width ϵ in which the drug concentration (C_M) is transported across the space via diffusion (at a rate D_M) with Dirichlet boundary conditions:

$$\frac{\partial C_M}{\partial t} = \frac{D_M}{r^2}\frac{\partial}{\partial r}\left(r^2 \frac{\partial C_M}{\partial r}\right) \approx 0, \tag{S9}$$

$$C_M = C_I = \text{constant}, \qquad r = 1, \tag{S10}$$

$$C_M = C_E = \text{constant}, \qquad r = 1 + \epsilon. \tag{S11}$$

We can solve equation (S9) via integration to give

$$C_M = -\frac{A}{r} + B, \tag{S12}$$

where A and B are constants that are determined using the boundary conditions (S10)-(S11) such that

$$A = -\frac{1+\epsilon}{\epsilon}(C_E - C_I), \qquad B = C_I - \frac{1+\epsilon}{\epsilon}(C_E - C_I), \tag{S13}$$

Therefore, by substitution into (S12), we acquire the solution

$$C_M = C_I + \frac{1+\epsilon}{\epsilon}\left(\frac{1}{r} - 1\right)(C_E - C_I) \tag{S14}$$

$$\approx C_I + \frac{1}{\epsilon}\left(\frac{1-r}{r}\right)(C_E - C_I), \tag{S15}$$

for $\epsilon \ll 1$. The inward flux at the cell membrane can then be found by Fick's law:

$$J = -D_M \frac{\partial C_M}{\partial r} = -D_M \frac{1}{\epsilon r^2}(C_E - C_I) = \frac{D_M}{\epsilon}(C_I - C_E)\,. \qquad r = 1\,. \tag{S16}$$

where $D_M/\epsilon = Q$ represents the permeability coefficient, proportional to the rate of intra-membrane aqueous diffusion and inversely proportional to the thickness of the membrane. Since $\epsilon \ll 1$ we can show that the outer membrane flux can also be derived such that

$$J = -D_M \frac{\partial C_M}{\partial r} = -D_M \frac{1}{\epsilon + 2\epsilon^2 + \epsilon^3}(C_E - C_I) \approx \frac{D_M}{\epsilon}(C_I - C_E)\,. \qquad r = 1 + \epsilon\,, \tag{S17}$$

and the inner and outer fluxes are equal as $\epsilon \to 0$ and thus,

$$D_I \frac{\partial C_I}{\partial r} = Q(C_E - C_I) = D_E \frac{\partial C_E}{\partial r}\,, \qquad r = 1\,. \tag{S18}$$

1.1.1 Numerical solution

We can solve the system numerically using the method of lines and gears whereby the following finite difference approximations in the spatial dimension reduce our PDE problem to an ODE problem. We apply central difference formulae for 1st and 2nd order spatial derivatives:

$$\frac{\partial C(r,t)}{\partial r} = \frac{C(r + \Delta r, t) - C(r - \Delta r, t)}{2\Delta r}\,, \tag{S19}$$

$$\frac{\partial^2 C(r,t)}{\partial r^2} = \frac{C(r + \Delta r, t) - 2C(r,t) + C(r - \Delta r, t)}{(\Delta r)^2}\,. \tag{S20}$$

Therefore, we can re-write our PDE model for interior dynamics in equation (S4) as

$$\frac{\partial C}{\partial t} = \frac{1}{r^2}\frac{\partial}{\partial r}\left(r^2 \frac{\partial C}{\partial r}\right) - \frac{V_{max} C}{C + K_m} = \frac{\partial^2 C}{\partial r^2} + \frac{2}{r}\frac{\partial C}{\partial r} - \frac{V_{max} C}{C + K_m}\,, \tag{S21}$$

$$\Rightarrow \frac{dC_i}{dt} = \frac{1}{\Delta r}\left(\frac{C_{i+1} - 2C_i + C_{i-1}}{\Delta r} + \frac{C_{i+1} - C_{i-1}}{r}\right) - \frac{V_{max} C_i}{C_i + K_m}\,, \tag{S22}$$

for $i = 1..R$ $(r = 0..1)$ where we use notation $C_{i+1} = C(r + \Delta r, t)$ such that an increase by 1 in subscript i corresponds to a radial increment in Δr as defined by the discretisation of the mesh (spatial domain). Similarly our PDE model for exterior dynamics is reduced to

$$\frac{dC_i}{dt} = \frac{D}{\Delta r}\left(\frac{C_{i+1} - 2C + C_{i-1}}{\Delta r} + \frac{C_{i+1} - C_{i-1}}{r}\right), \tag{S23}$$

for $i = R + 1..N + 1$ $(r = 1 + \Delta r..r_{max})$. We now inspect our boundary conditions in order to determine special case boundary values.

1.1.1.1 *Sphere centre boundary*

In the case where $i = 1$ we have

$$\frac{dC_1}{dt} = \frac{1}{\Delta r}\left(\frac{C_2 - 2C_1 + C_0}{\Delta r} + \frac{C_2 - C_0}{r}\right) - \frac{V_{max}C_1}{C_1 + K_m}, \tag{S24}$$

and therefore a singularity at $r = 0$. In the limit,

$$\frac{\partial C(0,t)}{\partial t} = \lim_{r\to 0}\left(\frac{\partial^2 C}{\partial r^2} + \frac{2}{r}\frac{\partial C}{\partial r} - \frac{V_{max}C}{C + K_m}\right) = 3\frac{\partial^2 C}{\partial r^2} - \frac{V_{max}C}{C + K_m}, \tag{S25}$$

by use of the Neumann boundary condition in equation (S6) and L'Hôpitals rule. Consequently,

$$\frac{dC_1}{dt} = \frac{3}{\Delta r}\left(\frac{C_2 - 2C_1 + C_0}{\Delta r}\right) - \frac{V_{max}C_1}{C_1 + K_m}. \tag{S26}$$

Thus we need to determine the value of the node C at $i = 0$, i.e. $r = 0 - \Delta r$. In order to do this we apply the Neumann boundary condition in equation (S6).

$$\frac{\partial C_1}{\partial r} = \frac{C_2 - C_0}{2\Delta r} = 0\,, \qquad r = 0\,, \tag{S27}$$

$$\Rightarrow C_0 = C_2\,. \tag{S28}$$

Therefore,

$$\frac{dC_1}{dt} = \frac{6}{(\Delta r)^2}(C_2 - C_1) - \frac{V_{max}C_1}{C_1 + K_m}, \tag{S29}$$

1.1.1.2 *Phase interface boundary*

For the interface boundary of the sphere we have a discontinuity and the following equations for the concentrations either side of the boundary:

$$\frac{dC_R}{dt} = \frac{1}{\Delta r}\left(\frac{\tilde{C}_{R+1} - 2C_R + C_{R-1}}{\Delta r} + \frac{\tilde{C}_{R+1} - C_{R-1}}{r}\right) - \frac{V_{max}C_R}{C_R + K_m}, \tag{S30}$$

$$\frac{dC_{R+1}}{dt} = \frac{D}{\Delta r}\left(\frac{C_{R+2} - 2C_{R+1} + \tilde{C}_R}{\Delta r} + \frac{C_{R+2} - \tilde{C}_R}{r}\right), \tag{S31}$$

where special boundary values have been highlighted with accents for extra clarity (note in passive diffusion uninhibited by any membrane permeation, $\tilde{C}_{R+1} = \tilde{C}_R$).

When $i = R$, we define $C_{i+1} = \tilde{C}_{R+1}$ using the equal flux boundary condition (see equation (S8)) and one-sided finite difference approximations and consequently we have

$$D_I \frac{\partial C_I}{\partial r} = D_E \frac{\partial C_E}{\partial r}, \qquad r = 1, \tag{S32}$$

$$\Rightarrow \frac{\tilde{C}_{R+1} - C_R}{\Delta r} = D \frac{C_{R+1} - \tilde{C}_R}{\Delta r}, \tag{S33}$$

$$\Rightarrow \tilde{C}_{R+1} = D\left(C_{R+1} - \tilde{C}_R\right) + C_R . \tag{S34}$$

To determine $\tilde{C}_R$ we use the transport boundary condition in equation (S18):

$$D_I \frac{\partial C_I}{\partial r} = Q(C_E - C_I), \qquad r = 1, \tag{S35}$$

$$\Rightarrow D_I \frac{\tilde{C}_{R+1} - C_R}{\Delta r} = Q\left(\tilde{C}_R - C_R\right), \tag{S36}$$

$$\Rightarrow \tilde{C}_R = \frac{D_I}{Q} \frac{1}{\Delta r}\left(\tilde{C}_{R+1} - C_R\right) + C_R . \tag{S37}$$

Therefore, following substitution into equation (S34),

$$\tilde{C}_{R+1} = \frac{\left(D - \tilde{Q}\Delta r D + \tilde{Q}\Delta r\right)C_R + \tilde{Q}\Delta r D C_{R+1}}{\tilde{Q}\Delta r + D}, \tag{S38}$$

where $\tilde{Q} = Q/D_I$ and substituting equation (S38) into equation (S37),

$$\tilde{C}_R = \frac{\tilde{Q}\Delta r C_R + D C_{R+1}}{\tilde{Q}\Delta r + D}. \tag{S39}$$

1.1.1.3 External boundary

When $i = N$ we have

$$\frac{dC_N}{dt} = \frac{D}{\Delta r}\left(\frac{C_{N+1} - 2C_N + C_{N-1}}{\Delta r} + \frac{C_{N+1} - C_{N-1}}{r}\right), \tag{S40}$$

and we determine the value of C at $i = N + 1$, i.e. $r = r_{max}$. In order to do this, we apply the Dirichlet boundary condition in equation (S7):

$$C_E = C_{r_{max}} = C_{N+1}, \qquad r = r_{max}, \tag{S41}$$

Therefore,

$$\frac{dC_N}{dt} = \frac{D}{\Delta r}\left(\frac{C_{r_{max}} - 2C_N + C_{N-1}}{\Delta r} + \frac{C_{r_{max}} - C_{N-1}}{r}\right). \tag{S42}$$

1.1.2 Model simulations

The systems of ODEs derived in section 1.1.1 were numerically integrated using MATLAB R2017b software to illustrate typical solutions (parameters provided in the legend for Figure 1) and a mesh of sufficient resolution to achieve steady state properties ($\frac{1}{r_{res}}\sum_r|C(t_{end},r) - C(0.9t_{end},r)| < 1 \times 10^{-4}C_{r_{max}}$ where r_{res} is the number of spatial steps in the grid and t_{end} is the final time-point value) and consistent internal accuracy ($\max\left(\frac{1}{r_{res}}\sum_r|C_{Ores}(t,r) - C_{Hres}(t,r)|\right) < 1 \times 10^{-4}C_{r_{max}}$ where $C_{Ores}(t,r)$ is the model solution at the original/default resolution (1001x1001 mesh) and $C_{Hres}(t,r)$ is the model solution at an increased mesh resolution where the spatial discretisation is increased 10-fold).

1.2 Carrier-mediated transport

We here reiterate a brief derivation of the simple carrier model used to derive the membrane flux boundary condition for clarity. It is assumed that the drug substrate (external, C_E, or internal, C_I) can reversibly bind to the transporter (facing the exterior, T, or interior, T', of the cell), with first order, mass-action kinetics. The bound transporter complexes are given by the variables CT and CT' for outward and inward facing transporters respectively. It is also assumed that the transporter (bound or unbound) undergoes a conformational change with first order kinetics to change position such that the binding site is facing either the exterior or interior of the cell (see schematic in Fig1A). The above processes are described in the system of ordinary differential equations below:

$$\frac{d[CT]}{dt} = k_{b1+}C_E[T] - k_{b1-}[CT] + k_{c1-}[CT'] - k_{c1+}[CT]\,, \tag{S43}$$

$$\frac{d[CT']}{dt} = k_{b2-}C_I[T'] - k_{b2+}[CT'] + k_{c1+}[CT] - k_{c1-}[CT']\,, \tag{S44}$$

$$\frac{d[T']}{dt} = k_{b2+}[CT'] - k_{b2-}C_I[T'] + k_{c2-}[T] - k_{c2+}[T']\,, \tag{S45}$$

$$\frac{d[T]}{dt} = k_{b1-}[CT] - k_{b1+}C_E[T] + k_{c2+}[T'] - k_{c2-}[T]\,, \tag{S46}$$

where k_c terms represent conformational changes, k_b terms represent binding/unbinding and the amount of receptor is conserved, i.e., $CT' + CT + T' + T = T_0$ (constant). The drug substrate flux is given by the difference between interior dissociation of bound substrate and the association of unbound substrate with inward facing receptors (and is equal to the difference between the association of unbound substrate to outward facing receptors and substrate dissociation at the cell exterior). Thus the flux is,

$$J = k_{b2+}[CT'] - k_{b2-}C_I[T'] = k_{b1+}C_E[T] - k_{b1-}[CT]\,, \tag{S47}$$

defined to be positive from outside to inside. It is assumed that the processes of binding and conformational changes are fast relative to the spatiotemporal drug concentration dynamics of the model at the cellular scale. Therefore we can find the steady state flux by setting the left-hand sides of equations (S43)-(S46) equal to 0 and solving to derive the 4 state variables in terms of C_E, C_I and the rate constants, subject to total receptor concentration, T_0. By substitution into equation (S47) we acquire the steady state flux:

$$J = \frac{T_0(C_E - \alpha_1 C_I)}{\alpha_2 + \alpha_3 C_E + \alpha_4 C_I + \alpha_5 C_E C_I}, \tag{S48}$$

where

$$\alpha_1 = \frac{k_{b1-}k_{c1-}k_{b2-}k_{c2-}}{k_{b1+}k_{c1+}k_{b2+}k_{c2+}}, \tag{S49}$$

$$\alpha_2 = \frac{(k_{c2+} + k_{c2-})(k_{b1-}k_{c1-} + k_{b1-}k_{b2+} + k_{c1+}k_{b2+})}{k_{b1+}k_{c1+}k_{b2+}k_{c2+}}, \tag{S50}$$

$$\alpha_3 = \frac{k_{b1+}\big(k_{b2+}(k_{c1+} + k_{c2+}) + k_{c2+}(k_{c1+} + k_{c1-})\big)}{k_{b1+}k_{c1+}k_{b2+}k_{c2+}}, \tag{S51}$$

$$\alpha_4 = \frac{k_{b2-}\big(k_{c1-}(k_{b1-} + k_{c2-}) + k_{c2-}(k_{b1-} + k_{c1-})\big)}{k_{b1+}k_{c1+}k_{b2+}k_{c2+}}, \tag{S52}$$

$$\alpha_5 = \frac{k_{b1+}k_{b2-}(k_{c1+} + k_{c1-})}{k_{b1+}k_{c1+}k_{b2+}k_{c2+}}. \tag{S53}$$

Therefore, we define the following flux boundary condition for the carrier-mediated transport model scenario:

$$D_I \frac{\partial C_I}{\partial r} = \frac{T_0(C_E - \alpha_1 C_I)}{\alpha_2 + \alpha_3 C_E + \alpha_4 C_I + \alpha_5 C_E C_I}, \qquad r = 1. \tag{S54}$$

1.2.1 Numerical solution

The carrier-mediated transport model was solved numerically using finite difference approximations as before. However, the new boundary condition in equation (S54) required the introduction of modified boundary values in the ODE approximations at the phase interface boundary,

$$\frac{dC_R}{dt} = \frac{1}{\Delta r}\left(\frac{\tilde{C}_{R+1} - 2C_R + C_{R-1}}{\Delta r} + \frac{\tilde{C}_{R+1} - C_{R-1}}{r}\right) - \frac{V_{max}C_R}{C_R + K_m}, \tag{S55}$$

$$\frac{dC_{R+1}}{dt} = \frac{D}{\Delta r}\left(\frac{C_{R+2} - 2C_{R+1} + \tilde{C}_R}{\Delta r} + \frac{C_{R+2} - \tilde{C}_R}{r}\right), \tag{S56}$$

With terminology analogous to the passive diffusion model, we use one-sided finite difference approximations and the carrier-mediated flux boundary condition in equation (S54) to determine $\tilde{C}_R$:

$$D_I \frac{\partial C_I}{\partial r} = \frac{T_0(C_E - \alpha_1 C_I)}{\alpha_2 + \alpha_3 C_E + \alpha_4 C_I + \alpha_5 C_E C_I}, \qquad r = 1, \tag{S57}$$

$$\Rightarrow \frac{\tilde{C}_{R+1} - C_R}{\Delta r} = \frac{\tilde{T}_0\left(\tilde{C}_R - \alpha_1 C_R\right)}{\alpha_2 + \alpha_3 \tilde{C}_R + \alpha_4 C_R + \alpha_5 \tilde{C}_R C_R}, \tag{S58}$$

$$\Rightarrow \tilde{C}_R = \frac{\left(C_R - \tilde{C}_{R+1}\right)(\alpha_2 + \alpha_4 C_R) - \Delta r \tilde{T}_0 \alpha_1 C_R}{\left(\tilde{C}_{R+1} - C_R\right)(\alpha_3 + \alpha_5 C_R) - \Delta r \tilde{T}_0}, \tag{S59}$$

where $\tilde{T}_0 = T_0/D_I$. Therefore, following substitution into the equal flux equation (S34), we can derive a quadratic equation for $\tilde{C}_{R+1}$,

$$\begin{aligned} &\tilde{C}_{R+1}^2(\alpha_3 + \alpha_5 C_R) \\ &-\tilde{C}_{R+1}\left(2C_R(\alpha_3 + \alpha_5 C_R) + \Delta r\tilde{T}_0 + DC_{R+1}(\alpha_3 + \alpha_5 C_R) + D(\alpha_2 + \alpha_4 C_R)\right) \\ &+C_R\big((DC_{R+1} + C_R)(\alpha_3 + \alpha_5 C_R) + D(\alpha_2 + \alpha_4 C_R)\big) - \Delta r\tilde{T}_0(D\alpha_1 C_R - C_R - DC_{R+1}) \\ &= 0, \end{aligned} \tag{S60}$$

and numerically integrate the full system of ODEs as described in section 0.

2 Parameterisation of passive diffusion

2.1 Diffusion of small molecule drugs

From a sample data base of 321 drugs, we identified several important physicochemical properties including molecular weight and density (Kyffin, 2018). These values allowed us to formulate an estimated range of likely diffusion coefficients for a wide range of drugs with a physically accurate range of weights and densities by employing the Stokes-Einstein equation describing the diffusion of spherical particles through a liquid with low Reynolds number,

$$D = \frac{k_B T}{6\pi\mu r}, \tag{S61}$$

where k_B is Boltzmann's constant, T represents temperature (assumed to be the physiological value, 310.15 K), μ is viscosity (assigned as 6.913×10^{-4} kg m^{-1} s^{-1}, the dynamic viscosity of water at 310.15 K) and r is the particle radius (m). To calculate the radius, we assume drugs can be represented spherically and use MW and density (ρ) data:

$$r = \sqrt[3]{\frac{3}{4\pi}\frac{\text{MW}}{\rho}\frac{1}{6.02\times10^{23}}}\ \text{m}\,. \tag{S62}$$

By implementing the formulae in (S61) and (S62) for the drug data base (MW ~100-1,200; density ~0.6-2.6 g/m^3), we were able to identify a feasible diffusion coefficient range of approximately 5×10^{-10} to 1×10^{-9} m^2/s.

2.2 Permeability as a function of lipophilicity

Menochet et al. (2012a, 2012b) discovered a log-linear relationship between lipophilicity ($\text{LogD}_{7.4}$) and "passive diffusion clearance" (P_{diff}) for xenobiotic uptake in human and rat hepatocytes. $\text{LogD}_{7.4}$ can be defined as a partition coefficient measure of lipophilicity at a physiologically relevant pH (pH = 7.4). For example, Menochet et al. derived the following relationship for human hepatocytes:

$$\log P_{diff} = 0.6316 \times \text{LogD}_{7.4} - 0.3143\,. \tag{S63}$$

In the study, the uptake rate is defined as the slope of the linear regression of the intracellular concentration-versus-time plot after 2 minutes at 4 °C (dimensions: [A]/10^6 cells·1/[T], [A] = amount units, [T] = time units). At early times, we assume that passive diffusion can be represented by the following system where the rate constant k_{in} represents the transport of drug into the cell:

$$\frac{dA_{cell}}{dt} = k_{in} V_{med} C_0 \tag{S64}$$

where A_{cell} is the amount of drug (units of moles) in the cellular compartment (expressed per 10^6 cells) and C_0 is the substrate or media concentration (moles dissolved in V_{med} = 400 μL of media in the

Menochet et al. study), which we assume to be an approximate constant external supply at early times, i.e., equivalent to dose concentration. The passive diffusion clearance, P_{diff}, is defined as the slope of the uptake rate against concentration,

$$\text{Uptake rate} = P_{diff} C_{out} \,, \tag{S65}$$

for media substrate C_{out} and dimensions,

$$\frac{[\mathrm{A}]}{10^6 \text{ cells} \cdot [\mathrm{T}]} = \frac{[\mathrm{V}]}{10^6 \text{ cells} \cdot [\mathrm{T}]} \cdot \frac{[\mathrm{A}]}{[\mathrm{V}]} \,, \tag{S66}$$

where [V] = volume units. By comparison with equation (S64), at early times, we have

$$\text{Uptake rate} = P_{diff} C_0 = \frac{dA_{cell}}{dt} = k_{in} V_{med} C_0 \,, \tag{S67}$$

$$\Rightarrow P_{diff} = k_{in} V_{med} \,, \tag{S68}$$

where P_{diff} has units of μL/min/10^6 cells in the Menochet et al. study.

In order to translate this uptake-related parameter into our spatial model we must derive the total intracellular-amount dynamics by integrating over the cell volume. At early times in low temperatures (no metabolism) we have the following system for drug concentration and transport into a single cell:

$$\frac{\partial C}{\partial t} = \begin{cases} \dfrac{\partial C_{cell}}{\partial t} = \nabla \cdot (D_{cell} \nabla C_{cell}) & r \leq R \\ \dfrac{\partial C_{out}}{\partial t} = \nabla \cdot (D_{out} \nabla C_{out}) & r > R \end{cases} \tag{S69}$$

We can define the amount of intracellular drug in the cell as follows:

$$A_{cell} = \int_{V_{cell}} C_{cell} \, dV = 4\pi \int_0^R C_{cell} \, r^2 dr \,. \tag{S70}$$

To define the total uptake rate for the entire cell we integrate the intracellular concentration dynamics with respect to the volume of the cell,

$$\frac{dA_{cell}}{dt} = \int_{V_{cell}} \frac{\partial C_{cell}}{\partial t} dV = \int_{V_{cell}} \nabla \cdot (D_{cell} \nabla C_{cell}) \, dV = \int_S D_{cell} \nabla C_{cell} \cdot \mathbf{n} \, dS \,, \tag{S71}$$

by the divergence theorem for surface, S, i.e. the surface area of the cell of radius R. It follows that

$$\int_S D_{cell} \nabla C_{cell} \cdot \mathbf{n} \, dS = 4\pi R^2 D_{cell} \left. \frac{\partial C_{cell}}{\partial r} \right|_{r=R} . \tag{S72}$$

We have the following boundary condition (see equation (S18)) at $r = R$,

$$D_{cell}\frac{\partial C_{cell}}{\partial r} = Q(C_{out} - C_{cell}) \approx QC_0, \tag{S73}$$

at early times ($C_{out} \approx C_0$ and $C_{cell} \approx 0$) where Q represents the permeability coefficient in units of [L]/[T] where [L] = length units. Substituting this result back into equation (S72), for a single cell, we obtain,

$$\frac{dA_{cell}}{dt} = 4\pi R^2 QC_0 = \frac{P_{diff}C_0}{10^6}. \tag{S74}$$

Therefore,

$$\frac{P_{diff}C_0}{10^6} = 4\pi R^2 QC_0\,, \tag{S75}$$

and we can derive the permeability coefficient for our model, Q, as a function of P_{diff}, itself a function of the physicochemical property $\text{LogD}_{7.4}$, and the radius of the cell, R:

$$Q = \frac{P_{diff}}{4\pi R^2} = \frac{1}{10^6}\frac{10^{(0.6316\times\text{LogD}_{7.4}-0.3143)}}{4\pi R^2}. \tag{S76}$$

3 Translating the multiscale model to a simple continuum model

3.1 Optimisation of effective parameters for the simple continuum model

The average steady state profiles for the full, multiscale models were acquired by extracting 8 1D radial profiles from cross-sections through the centre of the spheroid slice corresponding to the lines $y = 0$, $x = 0$, $y = x$ and $y = -x$ and calculating the mean. This method was initially validated by comparing the simple continuum model with the average radial profile of the full model with no intercellular space/zero porosity (i.e., the model used in Figure 4A), both using the default parameter set, i.e., $D_I^{Eff} = D_I$ and $Q^{Eff} = Q$ (see Figure S2A). In order to optimise the effective parameter values required to fit the simple continuum model to the cell-based models with inclusion of intercellular space, D_I^{Eff} and Q^{Eff} were varied by up to three orders of magnitude either side of the default dimensional value (e.g., $D_I^{Eff} = [1\times10^{-3}, 1\times10^{3}]\times D_I$ discretised over a log-scale for 51 points distributed within the interval) and the minimum residual error at steady state between the continuum and cell-based models was identified according to the following formula:

$$Error = \sum_i \left|\frac{C_{cont}(r_i, t^*) - \bar{C}_{cells}(r_i, t^*)}{\bar{C}_{cells}(r_i, t^*)}\right|, \tag{S77}$$

where C_{cont} represents the continuum model output, $\bar{C}_{cells}$ represents the average cell-based model output, r is radial distance (discretised at every µm from 0 to 750 µm, $i = 1{:}\,751$) and t^* indicates the

steady state. This process was repeated for both intercellular space geometries (wide and narrow) and a range of feasible permeability coefficients (corresponding to $\mathrm{LogD}_{7.4}$ = 1, 2, 3, 4, 5).

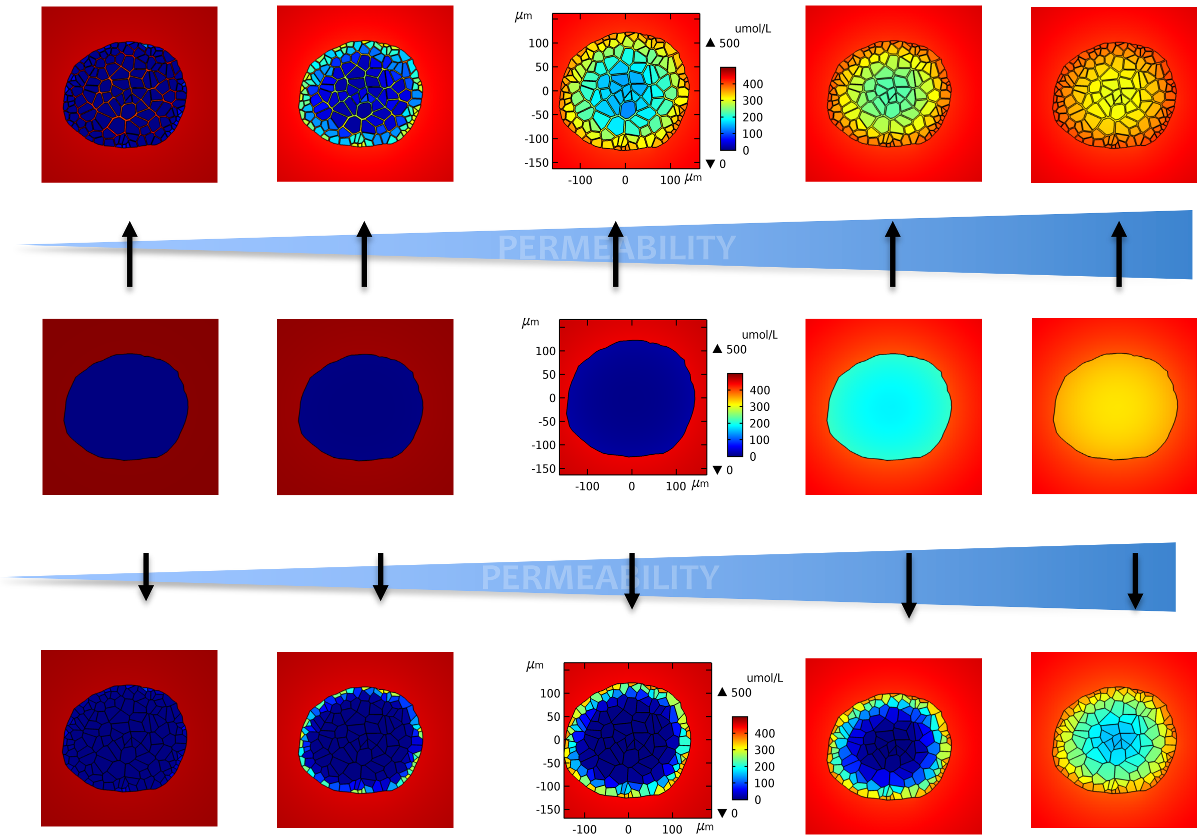

Figure S1: **Spatial distribution of drug concentration pre-optimisation.** The 2D spatial distribution of drug concentration at steady state for a range of permeabilities and intercellular space values are plotted. Permeability increases from left to right ($\mathrm{LogD}_{7.4}$ = 1, 2, 3, 4, 5). (Top row): Spatial plots for the wide intercellular space geometry. (Middle row): Spatial plots for zero intercellular space, analogous to the simple continuum model pre-optimisation. (Bottom row): Spatial plots for the narrow intercellular space geometry. All models solutions are generated using the default parameter set.

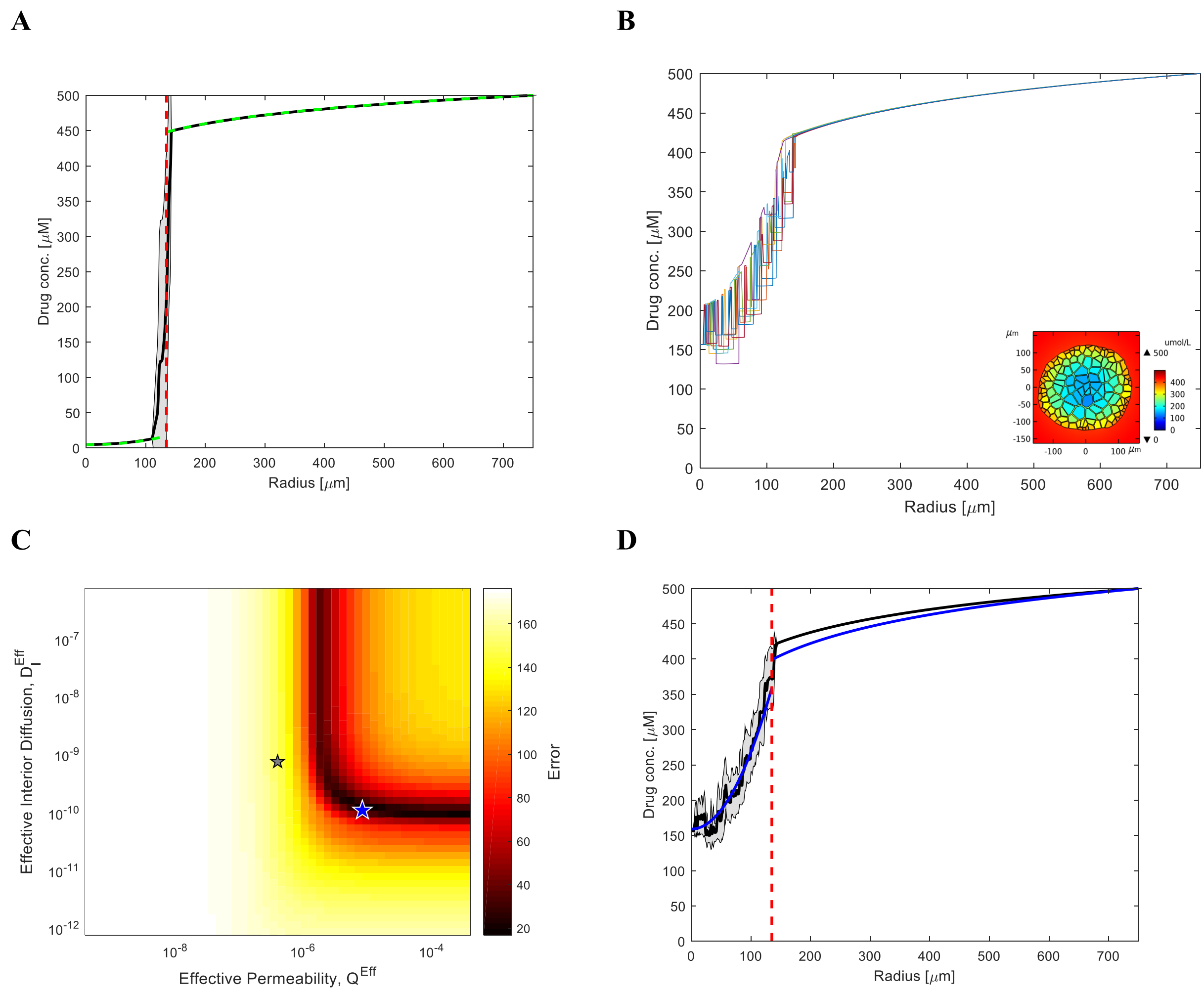

Figure S2: **Parameter optimisation for continuum model.** (A): Comparison of the "continuum model" output (green-dashed line, from equations 16-19 in the main manuscript) with the average radial profile for the "cell-based model" (black-solid line with grey standard deviation) with zero intercellular space (e.g., Figure 4A) at steady state using the same parameters (i.e., $D_I^{Eff} = D_I$ and $Q^{Eff} = Q$) The vertical red-dashed line indicates the spheroid boundary in the continuum model. (B): Example of 8 radial profiles used to calculate average behaviour of the cell-based model (default parameters at $LogD_{7.4}$ = 3 steady state). The 2D spatial distribution is also indicated (inset). (C): Example parameter sweep output representing error outputs (equation (S77)) for wide intercellular space, $LogD_{7.4}$ = 3. The original default parameters from the dimensional cell based model are indicated by the grey star. The minimum error representing optimal effective parameters is indicated by the blue star. (D): The continuum model steady state output (blue line) produced using the optimal effective parameters (i.e., blue star, (C)) is plotted against the average radial output of the cell-based model (black-solid line with grey standard deviation).

For plotting purposes in Figure 5F, cell-based models were compared with the continuum model by making use of the standard R^2 error metric:

$$R^2 = 1 - \frac{\sum_i \left| \frac{\bar{C}_{cells}(r_i, t^*) - C_{cont}(r_i, t^*)}{C_{cont}(r_i, t^*)} \right|}{\sum_i \left| \frac{\text{mean}\left(C_{cont}(r_i, t^*)\right) - C_{cont}(r_i, t^*)}{C_{cont}(r_i, t^*)} \right|}. \tag{S78}$$

References

Kyffin J A (2018). Establishing species-specific 3D liver microtissues for repeat dose toxicology and advancing in vitro to in vivo translation through computational modelling PhD Thesis, Liverpool John Moores University.

Ménochet K, Kenworthy K E, Houston J B and Galetin A (2012a) Use of mechanistic modeling to assess interindividual variability and interspecies differences in active uptake in human and rat hepatocytes. *Drug Metabolism and Disposition* 40: 1744-1756.

Ménochet K, Kenworthy K E, Houston J B and Galetin A (2012b) Simultaneous assessment of uptake and metabolism in rat hepatocytes: a comprehensive mechanistic model. *Journal of Pharmacology and Experimental Therapeutics* 341: 2-15.
