# Supplementary material for: Multiscale modelling of drug transport and metabolism in liver spheroids
Source: Interface Focus. 2020 Feb 14;10(2):20190041. doi: 10.1098/rsfs.2019.0041 (PMC7061947; doi:10.1098/rsfs.2019.0041)
Supplement: Supplementary Animations [file rsfs20190041supp2.pptx]

## Slide 1
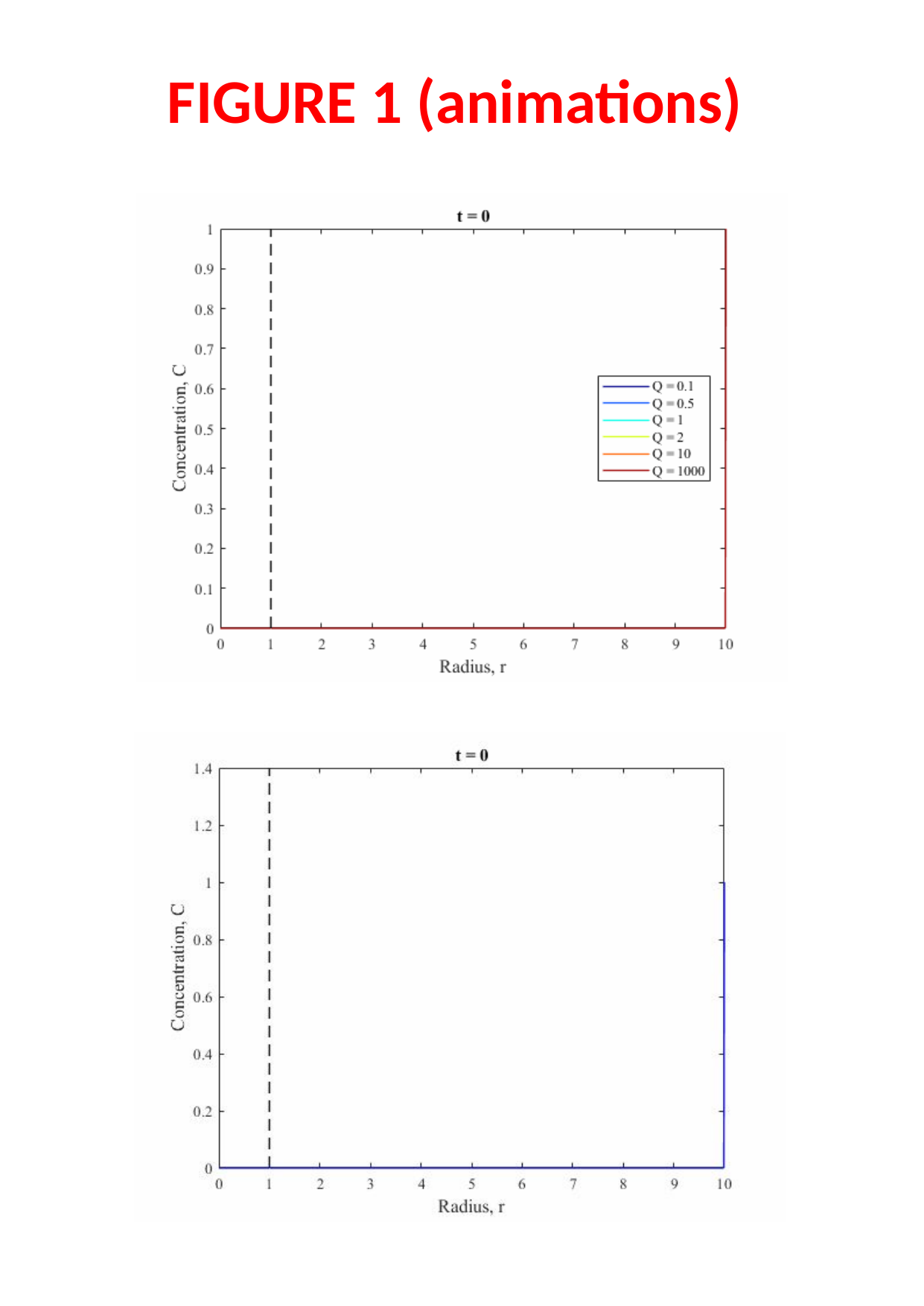

FIGURE 1 (animations)

## Slide 2
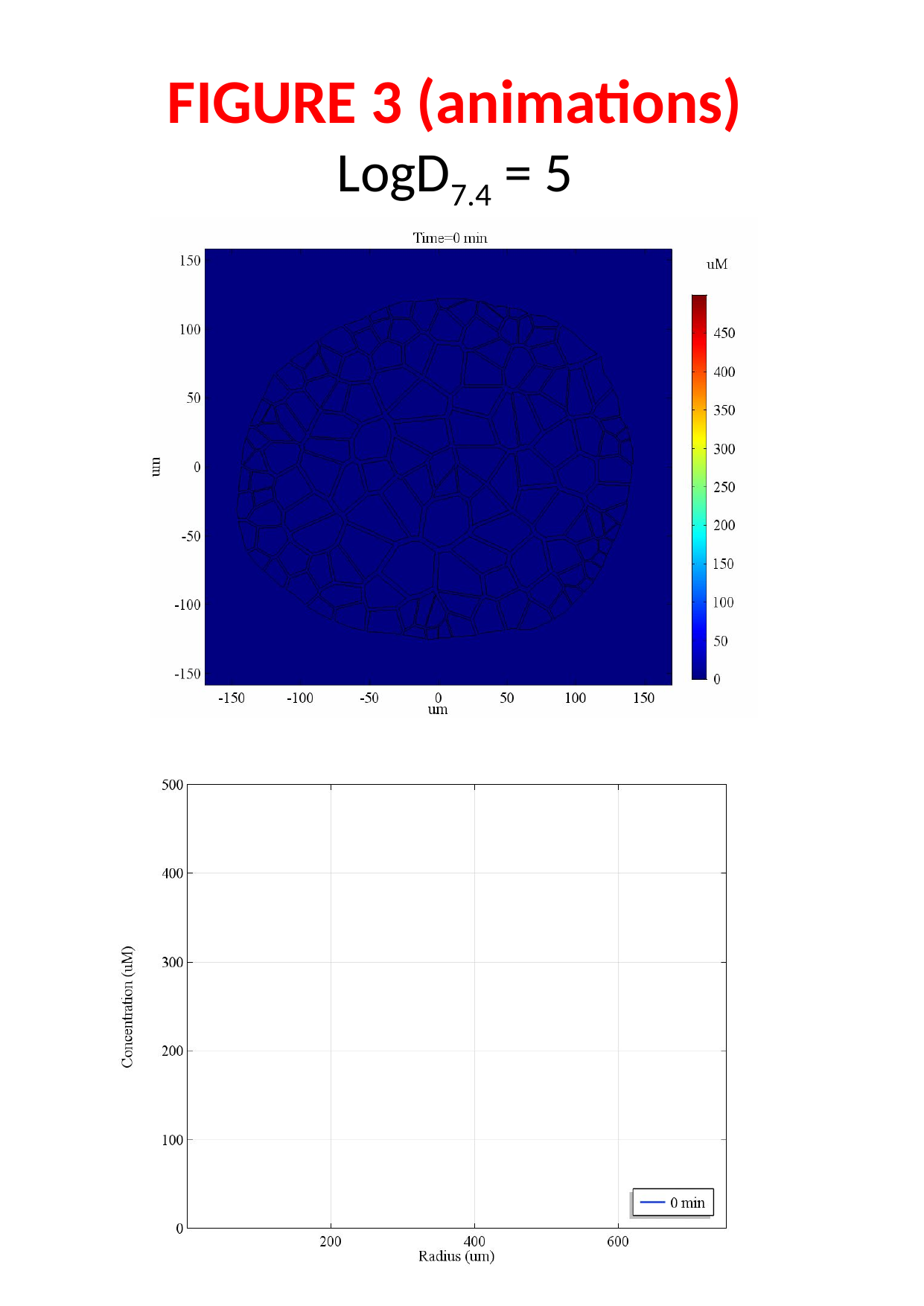

FIGURE 3 (animations)
LogD7.4 = 5

## Slide 3
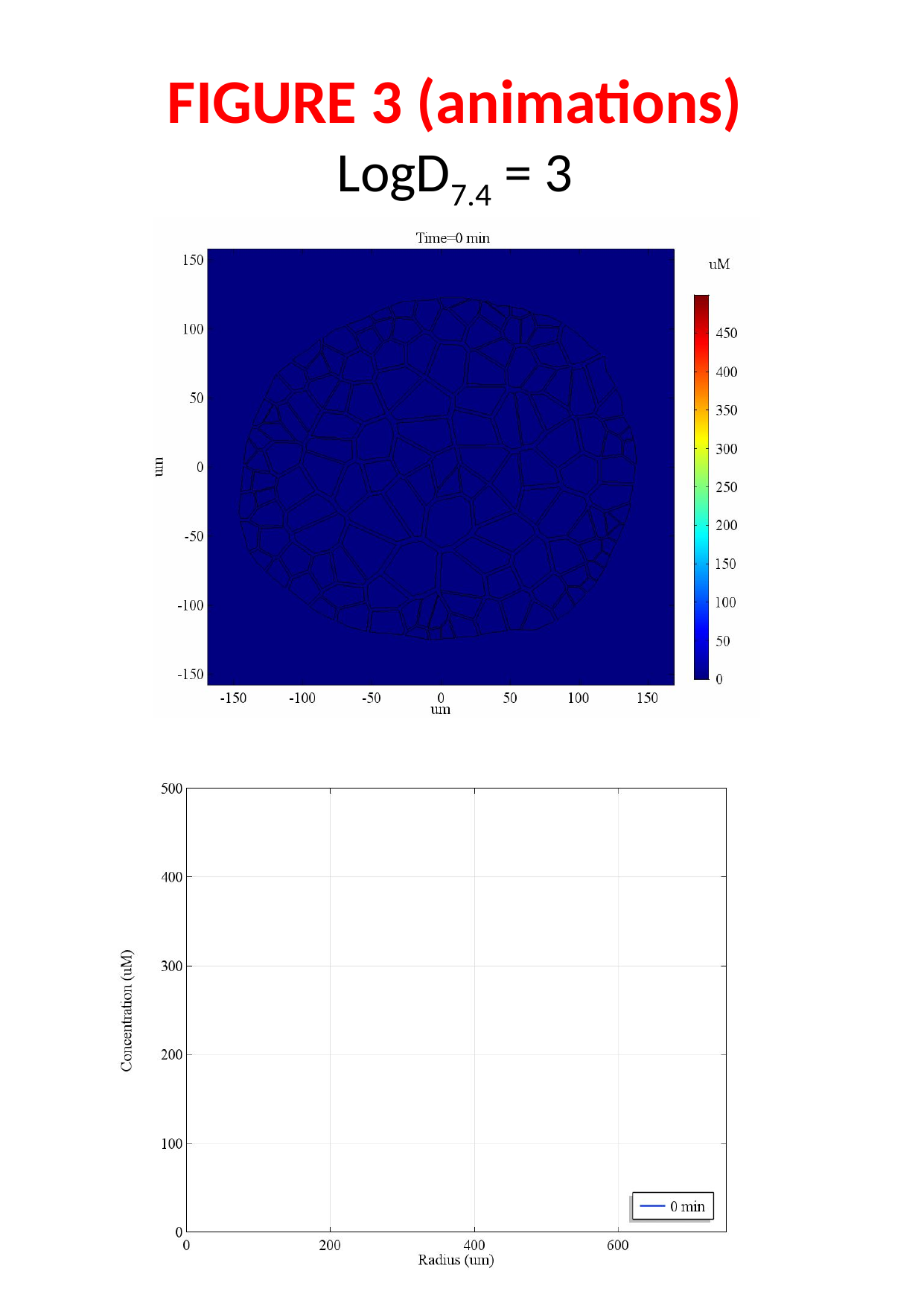

FIGURE 3 (animations)
LogD7.4 = 3

## Slide 4
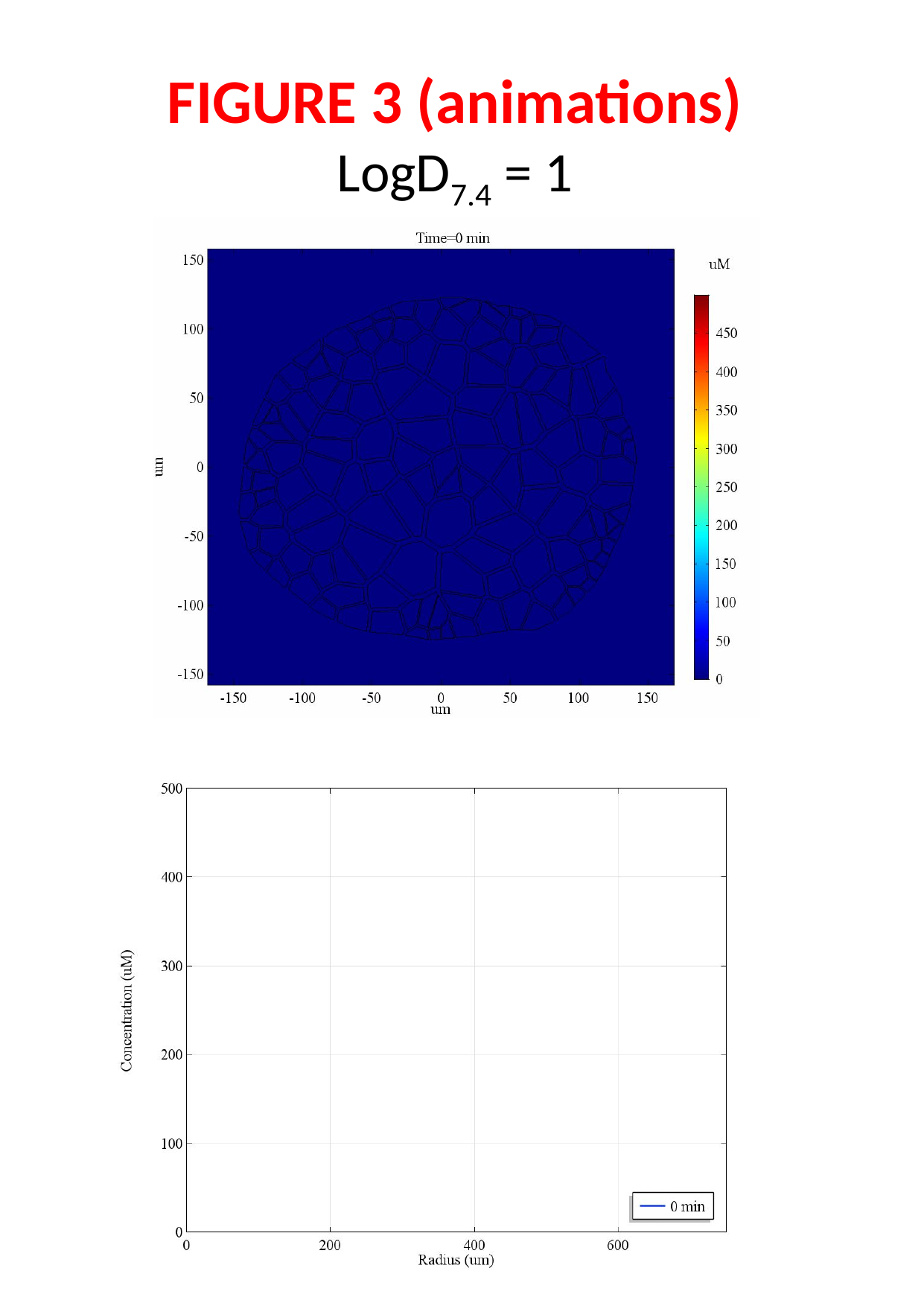

FIGURE 3 (animations)
LogD7.4 = 1
